# Molecular Momentum Transport at Fluid-Solid Interfaces in MEMS/NEMS: A Review

**DOI:** 10.3390/ijms10114638

**Published:** 2009-10-29

**Authors:** Bing-Yang Cao, Jun Sun, Min Chen, Zeng-Yuan Guo

**Affiliations:** 1 Key Laboratory for Thermal Science and Power Engineering of Ministry of Education, Department of Engineering Mechanics, Tsinghua University, Beijing 100084, China; E-Mails: mchen@tsinghua.edu.cn (M.C.); demgzy@tsinghua.edu.cn (Z.G.); 2 Institute of Nuclear and New Energy Technology, Tsinghua University, Beijing 100084, China; E-Mail: sunjun@tsinghua.edu.cn (J.S.)

**Keywords:** fluid-solid interfaces, molecular momentum transport, velocity slip, boundary conditions, momentum accommodation coefficient, micro/nanofluidics, molecular dynamics

## Abstract

This review is focused on molecular momentum transport at fluid-solid interfaces mainly related to microfluidics and nanofluidics in micro-/nano-electro-mechanical systems (MEMS/NEMS). This broad subject covers molecular dynamics behaviors, boundary conditions, molecular momentum accommodations, theoretical and phenomenological models in terms of gas-solid and liquid-solid interfaces affected by various physical factors, such as fluid and solid species, surface roughness, surface patterns, wettability, temperature, pressure, fluid viscosity and polarity. This review offers an overview of the major achievements, including experiments, theories and molecular dynamics simulations, in the field with particular emphasis on the effects on microfluidics and nanofluidics in nanoscience and nanotechnology. In Section 1 we present a brief introduction on the backgrounds, history and concepts. Sections 2 and 3 are focused on molecular momentum transport at gas-solid and liquid-solid interfaces, respectively. Summary and conclusions are finally presented in Section 4.

## 1. Introduction

### 1.1. Backgrounds

As predicted by the famous lecture “There’s Plenty of Room at the Bottom” delivered by Richard P. Feynman at the 1959 annual meeting of the American Physical Society [1], modern nanotechnologies have enabled the fabrication of many micro-/nano-electro-mechanical systems (MEMS/NEMS) with unique attributes, such as small mass, little energy dissipation and high accuracy and sensitivity [2–9]. Motors, actuators, sensors, reactors, pumps, valves, turbines, engines, etc. at nanometer to micrometer scales have been developed in recent years. Many of these micro-/nanodevices involve fluid and energy transports which are quite different from those at macroscale. Understanding the physics of fluid flows at micro-/nanoscale is crucial to designing, fabricating, utilizing and optimizing these MEMS and NEMS devices [10–23].

One of the most important characteristics of fluid flows at micro-/nanoscale is surface-dominated. The surface to volume ratio for a common machine with a characteristic length of 1 m is about 1 m^−1^, while that for a MEMS device with a size of 1 μm is 10^6^ m^−1^ and for a NEMS device having a length of 1 nm is 10^9^ m^−1^. The large surface to volume ratio for MEMS and NEMS devices enables factors related to surface effects to dominate the fluid flow physics at micrometer to nanometer scales [13,14,17,19,21]. Molecular behaviors at fluid-solid interfaces will play a dominant role in micro-/nanoscale mass, momentum and energy transports.

**Figure 1.**
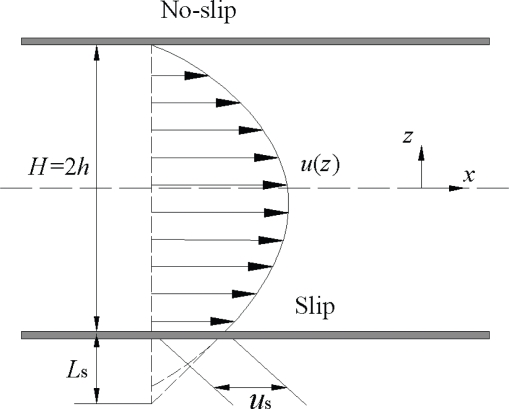
Schematic of plate Poiseuille flow considering no-slip and slip boundary conditions.

Let’s have a look at a simple example of a plate Poiseuille flow which considers the conventional no-slip and slip boundary conditions (BC) as shown in Figure 1, in which *u*_s_ is the slip velocity defined as the velocity difference between the solid and the fluid adjacent to the wall, *L*_s_ is the slip length, and *H* is the characteristic length of the Poiseuille flow system. The friction coefficient of the plate Poiseuille flow, which characterizes the flow drag, can be written as:

(1) $$f\;=\;\frac{48}{\text{Re}}\frac{1}{1+6\frac{L_{s}}{H}}$$

where Re is the Reynolds number [24]. It indicates that the effect of the boundary slip on the friction mainly depends on the ratio of the slip length to the characteristic length of the flow system. The slip length is comparable with the mean free path (MFP) for gases, about 0.065 μm for normal air [25], and is comparable with the molecular diameter for liquids, about 0.29 nm for water. The slip effect at fluid-solid interfaces on the friction can be ignored for macroscopic flows. However, it becomes very important for the micro-/nanoscale flows as the characteristic length decreases.

That is the reason why the present review paper is focused on molecular momentum transport at fluid-solid interfaces. Generally speaking, the boundary slip is only one of the apparent measurements of molecular momentum transport at fluid-solid interfaces, and is often used as boundary conditions for resolving Navier-Stokes (NS) equations. The details of molecular behavior are crucial in understanding flow physics at micro-/nanoscale and resolving particle-based equations in lattice Boltzmann (LB) [26] and direct simulation Monte Carlo (DSMC) [27] methods for modeling fluid mechanics. References [28–33] have reviewed the no-slip and slip conditions for liquid flows over solid surfaces. The present review, as a beneficial complement, covers molecular momentum transport at both gas-solid and liquid-solid interfaces, includes more recent achievements, and especially puts more emphasis on molecular behaviors near fluid-solid interfaces.

### 1.2. History

Studies on fluid mechanics at fluid-solid interfaces can be traced back to the early 19th century [34,35]. As early as in 1823, Navier pointed out that a fluid might slip on a solid surface, *i.e.*, there is a velocity difference, called slip velocity, between the fluid and solid at a fluid-solid interface [36]. He also introduced the idea of ‘slip length’ to quantify the slip boundary condition. Thus, the slip velocity *u*_s_ is linearly related to the slip length *L*_s_ and interfacial shear rate by”

(2) $$u_{s}\;=\;L_{s}\;{\left(\frac{\partial u}{\partial z}\right)}_{\text{interface}}$$

The linear slip boundary condition, called Navier’s slip model, is nowadays the standard characterization of velocity slip in fluid mechanics. In the following decades, there were debates about whether a fluid slipped on a solid or not. In the 1840s Stokes was commissioned by the Royal Academy of Science to investigate the true nature of the boundary conditions at fluid-solid interfaces and finally supported the no-slip image [37]. Poiseuille [38], Darcy [39] and Helmholtz [40] in their experiments confirmed the slip boundary condition that the velocity of a liquid adjacent to a solid surface was not always equal to that of the surface itself. However, Maxwell then pointed out that their experiments lacked necessary accuracy to distinguish such small slip length from a true no-slip boundary condition [41]. The following experiments offered by Maxwell [41], Whetham [42], Couette [43] and Ladenburg [44] came to the same conclusion that there was no evidence of slip. By the 1900s, though the concept of slip was still obscure, it was accepted that boundary slip, if it did exist, was too small to be observed.

In the mid-20th century there was no believable evidence for the slip at liquid-solid interfaces yet. Bearing in mind that the no-slip concept was doubtable [45,46], most fluid mechanics textbooks accepted the no-slip boundary condition, even without any acknowledgement of its empirical basis [34]. In the late 20th and early 21st centuries the rapid development in nanotechnology enabled more accurate detections and more effective molecular simulations [47–51] in fluid mechanics near fluid-solid interfaces. Nanometer scale slip lengths have been observed in recent years. We will review the recent advancements of the experimental and theoretical studies in Section 3.

The history of the study on the momentum transport at gas-solid interfaces is quite different compared with that at liquid-solid interfaces. In 1879 Maxwell [52] proposed a slip expression for gases over a solid surface based on kinetic theory:

(3) $$L_{s}\;=\;\frac{2-\sigma_{t}}{\sigma_{t}}\lambda$$

in which *σ_t_* is the tangential momentum accommodation coefficient (TMAC), *i.e.*, the fraction of molecules reflected diffusively from a solid surface, and *λ* is the mean free path of the gas molecules. In 1909 Knudsen did the first experiment and confirmed the Maxwell slip model [53]. In the following decades the Maxwell model was demonstrated to be valid for gases over solid surfaces by the Boltzmann transport theory [54–56] and experimental measurements [57–61]. The Maxwell model has been widely used in rarefied gas dynamics and gas microfluidics thus far. In recent years different slip models, such as higher order and nonlinear models, and various technologies for determining TMAC, which characterize molecular behaviors near gas-solid surfaces in more detail, have been developed. This is just what we will focus on in Section 2.

### 1.3. Molecular Momentum Transport and Boundary Conditions at Fluid-Solid Interfaces

**Figure 2.**
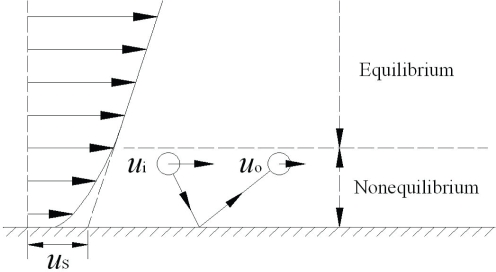
Schematic of velocity slip and nonequilibrium of molecules near a solid surface.

The momentum transport for molecules near a solid surface is in a nonequilibrium state as shown in Figure 2. The layer in a nonequilibrium state is about a mean free path in thickness adjacent to a solid surface. In rarefied gas dynamics this layer is often called Knudsen layer [57,62–64]. The incident molecules to the solid surface have a macroscopic velocity *u*_i_. During the collisions with the surface, the molecules will lose a fraction of the tangential momentum. Thus the reflected molecules have a different macroscopic velocity *u*_o_. *u*_i_ ≠ *u*_o_ because there are nearly no collisions between the incident and reflected molecules in this layer. The mean velocity of the incident and reflected molecules, *i.e.*, the fluid velocity, is (*u*_i_ + *u*_o_)/2. *u*_o_ can approach zero if the reflected molecules lose all their tangential momentum during the collisions with the solid surface. Even for this extreme case, the fluid velocity near the solid surface is *u*_o_/2, not zero. It is indicative of a velocity slip for fluids flowing over a solid surface.

**Figure 3.**
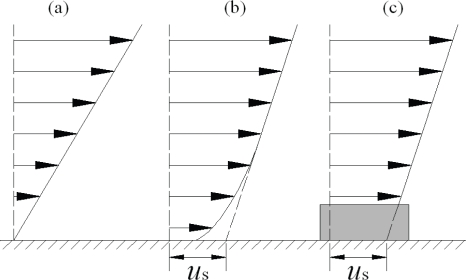
Schematic of (a) no-slip, (b) slip, and (c) effective (apparent) slip.

We often use three kinds of boundary conditions in micro/nanofluidics: no-slip, slip and effective (apparent) slip as shown in Figure 3: (a) No-slip: There is no velocity difference between the fluid and the wall at their interfaces. It remains an empirical assumption in classical fluid mechanics because early experiments were in good agreement with this boundary condition according to the foregoing review. (b) Slip: There is a velocity difference between the fluid and the wall at their interfaces, which can be characterized by the Navier’s linear slip model. It should be noted that the Navier’s model can be used only outside of the Knudsen layer. In the Knudsen layer Boltzmann transport equation has to be applied. (c) Effective (apparent) slip [31]: The effective (apparent) slip, being negative, zero or positive, is an equivalent concept of the macroscopic measurement of drag, force or flow rate etc. in experiments or simulations. Thus the effective slip is not an intrinsic slip but frequently used in hydrodynamics at liquid-solid interfaces. It may arise from the averaging effects of surface wettability, fluid viscosity, surface roughness, gas bubbles and other factors.

## 2. Gas-Solid Interfaces

The Boltzmann equations are usually good enough to describe the motions and interactions of gas molecules, especially for the rarefied gases since the gas density is much lower than the liquid density. The Knudsen number (Kn), which is defined as the ratio of the mean free path to the characteristic size in gas flows, is the dimensionless number to characterize the rarefaction:

(4) $$\text{Kn}\;=\;\frac{\lambda }{H}$$

in which *λ* is the mean free path of gas molecules, and *H* is the characteristic size in gas flows.

The gas-solid momentum transport is affected by both the interactions between nearby gas molecules (gas-gas interactions) and the interactions between gas molecules and solid atoms (gas-solid or gas-wall interactions) near the solid surface. The rate of momentum transport varies for different Knudsen numbers due to the change of relative importance of gas-gas and gas-wall interactions. According to the dimensionless Boltzmann equation [65], the intermolecular force weakens gradually at larger Knudsen numbers. The gas flows are generally divided into four regimes according to the Knudsen number [66]:

**Table:** 

Kn < 0.001	Continuum regime
0.001< Kn < 0.1	Slip regime
0.1< Kn < 10	Transition regime
Kn > 10	Free molecular regime

When Kn is smaller than 0.001, the Navier-Stokes equations with no-slip boundary condition are applicable. Seen from the definition in Equation (4), a larger Kn may arise from a longer mean free path, *i.e.*, rarefied gas flows, or a smaller system size, *i.e.*, microscale and nanoscale gas flows. When Kn is large, slip phenomena, *e.g.*, the velocity slip and temperature jump, appear in the thin gas layer adjacent to the solid wall, which is named as the Knudsen layer. Several papers, based on the Boltzmann equations [67], described the fluidics in Knudsen layer and found that its thickness was 0.9 to 4.9 times of the mean free path [68–72]. Inside the Knudsen layer, the momentum transport in high Kn flows is different from that in low Kn flows in two aspects [17,73]. In rarefied gas flows, where the mean free path is much larger, the continuum assumption is not valid; while for gas flows in microchannels and nanochannels, parameters related to wall effects become dominated since the ratio of area to volume is large. Therefore, proper models have to be applied to describe gas-wall interactions in high Kn flows. In the slip regime with moderate Knudsen numbers, the NS equations are also valid but slip boundary conditions should be taken into account. For larger Kn, the NS equations break down, and the Boltzmann equations or methods based on the kinetic theory should be applied. In this case, the Maxwell-type model or Cercignani-Lampis-Lord model are often used at the boundary [27,68].

In such boundary slip models, the accommodation coefficients are necessary inputs to characterize the gas-solid momentum transport. The tangential and normal momentum accommodation coefficients (abbreviated as TMAC and NMAC) are usually used to characterize the momentum exchanges parallel and perpendicular to the surface. Indeed, the early researches on gas-wall interactions were only focused on determining the accommodation coefficients in these boundary slip models [74]. Furthermore, the study of the accommodation coefficients also helps understanding the gas-solid momentum transport by investigating the velocity distributions of impinging and reflected gas molecules.

In the following sections, molecular motion is primarily introduced through various models. Then, the authors focus on the research on tangential momentum transport, including slip models and the TMAC. At last, some promising problems are discussed for further studies.

### 2.1. Description of Molecular Distributions

In researches on gas-wall interactions, theoretical models have been developed to show the velocity distribution relation between the incident and reflected gas molecules. Thus, the tangential and normal momentum transport can be expressed explicitly in such models.

The first and most fundamental description of gas-wall interactions was proposed by Maxwell in 1859 [75]. The gas-wall interactions were divided into two processes: incidence and reflection. In kinetic theory, Maxwell established two classical reflection models, *i.e.*, the diffusive and specular models. In the diffusive model, gas molecules are adsorbed near the wall for a long time and totally forget the incident information. After that, these molecules are desorbed and re-emitted to the half space above the wall in all angles equiprobably. The tangential momentum is completely lost and the normal momentum changes to fixed values related to the wall temperature (see Equation 35) in diffusive reflections. In the specular model, where gas molecules experience direct elastic collisions with the wall without adsorption, the tangential momentum holds and the normal momentum only changes the direction.

The diffusive model is widely used in practical applications, especially in macroscale and engineering problems, in which the adsorption time is also usually much longer than that in rarefied gas flows or in flows on extremely smooth surfaces. Under conditions where the adsorption time is not extremely long or short, the diffusive or specular model alone is not appropriate. Maxwell [52] combined the diffusive and specular models together, named Maxwell-type model, that one fraction of molecules are diffusively reflected while the other are specularly reflected. The combination of the two models makes the Maxwell-type model to be a single-parameter model, which can not describe the momentum and energy transport at the same time. Furthermore, the fraction of diffusively reflected molecules is an empirical parameter and difficult to be determined because it strongly depends on many physical factors at the gas-solid interfaces. Many researchers treated it as the TMAC in order to calculate the tangential momentum transport, while used the energy accommodation coefficient (EAC) for the energy transport. However, in most cases, the TMAC and EAC are not equal in the same problem. Moreover, the single parameter cannot describe the momentum transport in both the tangential and normal directions. In addition, the Maxwell-type model is only applicable for the gas flows where the rarefaction and roughness effects are not evident.

According to the reciprocal law, Cercignani and Lampis [76] developed a phenomenological model (CL model) to describe gas-wall interactions, in which two parameters related to the tangential momentum and normal energy transport were introduced. Later, Lord [77,78] modified and extended the CL model (named as CLL model) in the direct simulation Monte Carlo method (DSMC) [27] and made it widely used in theoretical and numerical researches of rarefied gas flows. The CLL model improves the velocity distributions of reflected gas molecules and agrees well with the lobular distribution but does not agrees well with the results observed by molecular beam experiments [79].

There are also some models describing the gas-wall interactions by fitting with the experimental results based on introducing some empirical parameters, in which the Nocilla model and multi-flux model are two of the most typical ones. In the Nocilla model [80,81], the reflected speed ratio, reflection angle and some other parameters can be well selected so as to agree with the computational results [82]. Nevertheless, Collins and Knox [83] implied that two parameters in this model could not be chosen arbitrarily and were related to the TMAC and NMAC. In the multi-flux model [84], the phase space of incident molecules is divided into several regimes according to the incident velocities and angles. By the combination of the diffusive model and CLL model with different possibilities, the multi-flux model can be also comparable with other models by selecting proper possibilities, but it is too specific for practical applications.

Since the interactions at the gas-solid interfaces are complicated and strongly depend on the surface conditions, models with limited parameters cannot fully describe the reflection of gas molecules yet. The multi-stage model [79], based on molecular dynamic simulations, could determine the information of reflected gas molecules in three stages. Using the multi-stage model, the DSMC results agreed well with the molecular beam experiments and improved the CLL model significantly.

From the above review of models about gas-wall interactions, the descriptions of reflected gas molecules by the surfaces are not good enough due to the complicated situations at gas-solid interfaces. More parameters are required to be added into the models and more realistic models have to be established in the future.

### 2.2. Tangential Momentum Transport

Besides the descriptions of molecular distributions, models and parameters of macroscopic properties are usually concerned in researches of momentum transport, especially in tangential momentum transport. Tangential momentum transport is one of the most important points because it is often related to flow resistance on surfaces, mass and volume flow rates, as well as pressure drop in channels and tubes. The velocity profiles inside the Knudsen layer also drew much attention. Many experts tried to employ slip models as boundary conditions in resolving the NS equations and extended the applicability of the NS equations to larger range of Knudsen numbers. In addition, the TMAC is also the key input in the slip models.

#### 2.2.1. Slip Models

Since the Knudsen layer is only several mean free paths in thickness, there are very few gas molecules in this thin layer, and the continuum hypothesis is not valid. As the Knudsen number increases, the Knudsen layer gradually extends from near-surface to the main flow so that the NS equations with traditional no-slip boundary conditions are not proper to describe the slip phenomena for large Kn. Higher order equations should be used, such as the Burnett equations [85], super-Burnett equations [86], Grad 13 moment method [72] and normalized Grad moment methods [87], but there are still many problems in the boundary conditions and computational stability. The Boltzmann equations are good enough for rarefied gas flows, while the direct solutions of the Boltzmann equations are much limited due to the strong nonlinear collision term. Moreover, the atomic simulations (molecular dynamics or Monte Carlo method) can trace the molecular motions and obtain more detailed results. However, the computation power of computers is not strong enough, and the space and time scales in atomic simulations are confined in small systems and time ranges. Therefore, in order to describe the phenomena inside the Knudsen layer, a more practical and compromise way is to find appropriate boundary models to extend the applicability of the NS equations. And it has been reported that the NS equations with modified slip models showed good results in the slip regime of 0.001 < Kn < 0.1.

As a common phenomenon in tangential momentum transport, the velocity slip can be described by models based on and developed from the Maxwell-type model. Maxwell [52] assumed that one half of the gas molecules near the wall are incident and the other half are reflected, in which one part of them is diffusive while the rest is specular. Using Taylor series and omitting high order terms, the famous Maxwell first-order slip model is:

(5) $$u_{s}\;=\;{\frac{2-\sigma_{t}}{\sigma_{t}}\lambda \frac{du}{dz}|}_{w}$$

where *u_s_* is the velocity slip on the wall surface, *i.e.*, the difference between gas velocity adjacent to wall and the wall velocity, *σ* *_t_* denotes the TMAC at the wall, $\frac{2-\sigma_{t}}{\sigma_{t}}$ is generally named the slip coefficient and is usually studied along with the TMAC together [88], and ${\frac{du}{dz}|}_{w}$ is the normal gradient of gas velocity at the wall. In addition, velocity slip is also affected by thermal creep or thermal transpiration, which is not considered in isothermal flows in this review.

From Equation (5), the velocity slip is related to the TMAC, the mean free path or Knudsen number, the velocity gradient above the wall, and their combination or expression. Therefore, researches on slip models always concern on the accuracy of these parameters and reasonable expressions.

Slip models can be divided into two categories: the linear and nonlinear models. The Maxwell model of Equation (5) is the earliest and most widely used linear slip model, in which the velocity slip is proportional to the mean free path and the constitute relation is linear. Researches on linear slip models are all based on and developed from the Maxwell model, and care about the magnitude and expression of the slip coefficient [66,68,75,89–93]. While in nonlinear slip models, the constitute relations are nonlinear and the velocity slip is no longer linear with the mean free path.

##### 2.2.1.1. Linear Slip Models

In slip models, the velocity slip is proportional to the mean free path and velocity gradient. In other words, the constitute relation is linear. Besides the Maxwell first-order expression in Equation (5), the linear slip models are usually used in a second-order form:

(6) $$u_{s}\;=\;{C_{1}\lambda \frac{du}{dz}|}_{w}\;-\;{C_{2}\lambda^{2}\frac{d^{2}u}{dz^{2}}|}_{w}$$

in which *C*_1_ and *C*_2_ are first- and second-order slip coefficients, respectively. For Maxwell model in Equation (5), $C_{1}\;=\;\frac{2-\sigma_{t}}{\sigma_{t}}$ and *C*_2_ = 0.

Research on linear slip models is concerned with the slip coefficients in two aspects: their magnitudes when *σ_t_* =1 and their expressions, especially in the form of the TMAC. When *σ_t_* =1 for the diffusive boundary condition, the first- and second-order slip coefficients from literature are listed in Table 1, in which the results are not consistent. The first-order slip coefficients are almost around unity while the second-order slip coefficients range from −0.5 to larger than one. In experiments, the second-order slip coefficients being derived from mass flux would be underestimated for about 0.3 without considering the effect of the Knudsen layer [93]. For instance, Maurer *et al*. [94] tested the gas flows in microchannels and found that the second-order slip coefficient for N_2_ was 0.26 ± 0.1, while Sreekanth [95,96] gave 0.14.

The investigation on the expression of slip coefficients is much difficult, and most expressions are based on the Maxwell model, as shown in Table 2. The first-order slip coefficients are related to the TMAC while the second-order slip coefficients are quite different.

**Figure 4.**
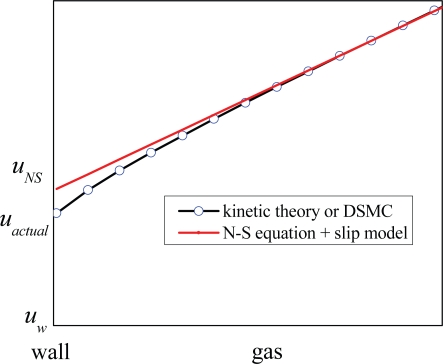
Comparison of velocity profiles by kinetic theory and linear slip theory.

In linear slip models, the velocity slip is linear with the mean free path, in which the mean free path is constant along the channel’s height between two walls. The NS equations with linear slip models cannot describe the flow inside the Knudsen layer accurately, especially for large Kn gas flows, as shown in Figure 4. For the region far from the wall, the velocity profile predicted by NS equation with linear slip models agrees well with the results by the kinetic theory or DSMC method. While in the near wall region, the velocity profile predicted by the NS equations is different from that by the kinetic theory inside the Knudsen layer, and the velocity slip predicted by linear slip models (*u_NS_* , also called fictitious velocity slip [102]) is larger than that by the kinetic theory (*u_actual_*, the actual velocity slip). Schram [103] deduced that $u_{NS}\;=\;\sqrt{2}u_{actual}$. If the velocity profile inside the Knudsen layer is truly obtained by modifying the slip coefficients in linear slip models, the velocity profile outside is not accurate any more [66,93,100]. Therefore, the linear slip models can only applicable for low Kn gas flows (Kn < 0.1) where the effect of the Knudsen layer reduces to a small region adjacent to the wall.

##### 2.2.1.2. Nonlinear Slip Models

Since the linear slip models ignore the wall effects on the mean free path and velocity profiles, the description of the Knudsen layer must be more accurate using nonlinear slip models, in which the velocity slip is not proportional to the mean free path any more. In nonlinear slip models, the constitutive relations are modified so that the stress is expressed in a more realistic way. The mean free path is also modified by effective expressions.

By replacing the viscosity (*μ*) and the velocity gradient (shear rate) in Equation (5) with the tangential stress (*τ*) [52,100]:

(7) $$\tau \;=\;-\mu \frac{\partial u}{\partial z}$$

The velocity slip related to the stress is:

(8) $$u_{s}\;=\;-\frac{2-\sigma_{t}}{\sigma_{t}}\lambda \frac{\tau }{\mu }$$

In complicated situations, the stress is not as simple as that in Equation (7). Lockerby *et al*. [100] revealed that the normal velocity of near-wall gas changed along the wall when the wall was not flat or the wall moved in the normal direction. The velocity slip in the tangential direction was affected by both the tangential and normal velocity and expressed as:

(9) $$u_{s}\;=\;\frac{2-\sigma_{t}}{\sigma_{t}}\lambda (\frac{\partial u_{x}}{\partial z}+\frac{\partial u_{z}}{\partial x})$$

in which *u*_x_ and *u*_z_ are, respectively, gas velocities in the tangential and normal directions. The prediction of the Couette flow between two coaxial cylinders could be improved by using the slip model of Equation (9) and agreed well with the DSMC results [100]. Einzel *et al*. [104] also setup a slip model considering the surface roughness above the wall.

In planar flows, Lockerby *et al*. [102] established the constitutive relation according to the wall function in turbulent flows and the solution to the linearized Boltzmann equation [68]:

(10) $$\tau \;=\;-\mu {\left(1+\frac{7}{10}{\left(1+\frac{z}{\lambda }\right)}^{-3}\right)}^{-1}\frac{du}{dz}$$

in which *z* denotes the distance from the wall. The corresponding slip model is:

(11) $$u_{s}\;=\;-\sqrt{\frac{2}{\pi }}\frac{2-\sigma_{t}}{\sigma_{t}}\lambda \frac{\tau }{\mu }$$

In Equation (10), the constitutive relation can be expressed by using the effective viscosity (*μ_eff_*) shown in Equation (12), in which the effective viscosity is apparently position-related and affected by the wall:

(12) $$\mu_{\text{eff}}\;=\;\mu {\left(1+\frac{7}{10}{\left(1+\frac{z}{\lambda }\right)}^{-3}\right)}^{-1}$$

The effective viscosity in Equation (12), fitted from the velocity profile near the wall, is not physically meaningful in further applications for the mean free path or the thermal conductivity [105,106]. Reese *et al.* [105] continued to use the scaled constitutive relation to extend the NS equations, and the stress and effective viscosity are:

(13) $$\tau \;=\;-\mu {\left[1-A({D\sigma }_{t}+E){\left(1+\hat{z}\right)}^{A-1}\right]}^{-1}\frac{du}{d\hat{z}}$$

(14) $$\mu_{\text{eff}}\;=\;\mu \;{\left[1-A({D\sigma }_{t}+E){\left(1+\hat{z}\right)}^{A-1}\right]}^{-1}$$

in which $\hat{z}\;=\;\frac{\sqrt{\pi }}{2}\;\frac{z}{\lambda }$ is the reduced distance from the wall, parameters *A* , *D* and *E* are calculated from resolving the Boltzmann equation using the hard sphere or the Bhatnagar-Gross-Krook (BGK) models [107]. Thus, the slip model is:

(15) $$u_{s}\;=\;-\frac{2-\sigma_{t}}{\sigma_{t}}\;\zeta \lambda \;\frac{\tau }{\mu }$$

in which *ζ* is about 0.8 and is close to $\sqrt{2/\pi }$ in Equation (11).

Following the first-order scaled constitutive relation of Equation (15), which was well applied in Kramer’s problem, Lockerby *et al.* [108] established a second-order scaled constitutive relation to simulate gas flows on sphere surfaces, and the slip model is:

(16) $$u_{s}\;=\;-0.798\lambda \frac{\tau }{\mu }-0.278\frac{\lambda^{2}}{\mu }\frac{d\tau }{dz}$$

**Figure 5.**
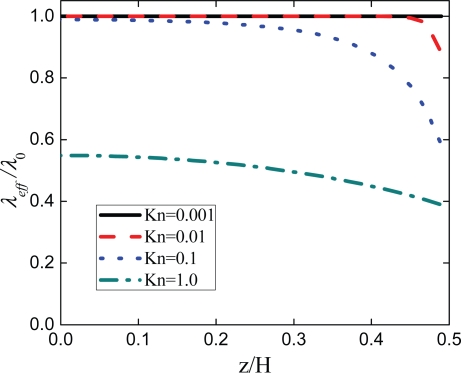
Effective mean free path distributions for different Knudsen numbers.

Besides the investigations on various constitutive relations, Stops [109] analyzed the space distribution of the mean free path between two parallel walls and introduced the notion of effective mean free path (*λ_eff_*) as:

(17) $$\lambda_{\text{eff}}\;=\;\lambda_{0}\left\{1\;+\;\frac{1}{2}[(a\;-\;1)e^{-a}\;+\;(b\;-\;1)e^{-b}\;-\;a^{2}E_{i}\;(a)\;-\;b^{2}E_{i}\;(b)]\right\}$$

(18) $$a\;=\;z/\lambda_{0}$$

(19) $$b\;=\;(H\;-\;z)/\lambda_{0}$$

(20) $$E_{i}(x)\;=\;\int_{1}^{\infty }t^{-1}e^{-xt}dt$$

in which *λ*_0_ is the mean free path of gas flows with no bounding wall, *z* is the distance from the wall, and *H* is the distance between two separated walls. For different Kn, the wall effect on the mean free path is quite different, and the Knudsen layers enlarge from the near wall region and may overlap for large Kn, as shown in Figure 5.

In kinetic theory, the mean free path is related to the viscosity for equilibrium rarefied gases by [68]:

(21) $$\lambda \;=\;\frac{\mu }{p}\sqrt{\frac{\pi RT}{2}}$$

If this relation is also valid for the effective mean free path and effective viscosity [110–112] in constant pressure (density) isothermal gas flows, the effective viscosity is:

(22) $$\mu_{\text{eff}}\;=\;\lambda_{\text{eff}}\frac{p}{\sqrt{\frac{\pi RT}{2}}}$$

(23) $$\mu_{\text{eff}}\;=\;\mu_{0}\left\{1+\frac{1}{2}[(a-1)e^{-a}+(b-1)e^{-b}-a^{2}E_{i}\;(a)-b^{2}E_{i}\;(b)]\right\}$$

in which *μ*_0_ is the viscosity without the wall effect.

Guo *et al.* [113] applied the effective viscosity of Equation (23) to the NS equations so that the isothermal Couette and Poiseuille flows can be described as:

(24) $$\frac{d}{dz}(\mu_{\text{eff}}\;\frac{du}{dz})\;=\;0$$

(25) $$\frac{d}{dz}(\mu_{\text{eff}}\;\frac{du}{dz})+\rho g_{x}\;=\;0$$

in which *g_x_* is the driving acceleration in isothermal Poiseuille flows. According to the effective mean free path in Equation (17), Guo *et al*. [110] setup a second-order slip models as below, and extended the applicability of the NS equations to higher Kn (up to about 4) gas flows, in which the mass flux agreed well with experimental results in Refs. [114,115]:

(26) $$u_{s}\;={C_{1}(\lambda_{\text{eff}}\;\frac{du}{dz})|}_{w}\;-\;{C_{2}\left[\lambda_{\text{eff}}\;\frac{d}{dz}(\lambda_{\text{eff}}\;\frac{du}{dz})\right]|}_{w}$$

(27) $$C_{1}\;=\;\frac{2-\sigma_{t}}{\sigma_{t}}(1-0.1817\sigma_{t})$$

(28) $$C_{2}\;=\;\frac{1}{\pi }\;+\;\frac{1}{2}C_{1}^{2}$$

Arlemark *et al*. [111,112] used Simpson’s numerical integration involving several subintervals to replace the exponential integral in Equation (20) and obtained the similar results as Stops’ in Ref. [109]. Furthermore, Arlemark *et al*. [111,112] compared the velocity profiles predicted by the linearized Boltzmann equations with that by the NS equations through altering the slip coefficients in Equation (26), and found the best slip coefficients for Kn < 0.113 were *C*_1_ = 1 in first-order slip models while *C*_1_ = 0.05 and *C*_2_ = 0.63 in second-order slip models.

The nonlinear flows inside the Knudsen layer are still difficult to describe, whether for the constitutive relations or for the effective mean free path and viscosity. The constitutive relations from the numerical results of the Boltzmann equations are often lack of physical explanations, and the relation between the effective mean free path and viscosity are not valid when the density (pressure) are not constant in the microchannels [116,117]. Therefore, researches on nonlinear slip models, especially for large Kn gas flows, are still challenging.

#### 2.2.2. TMAC

In aforementioned slip models, the TMAC is one of the most important parameters to characterize the tangential momentum transport between the gas and wall. The momentum accommodation coefficients were first proposed by Knechtel and Pitts [118] to investigate the surface gas dynamics in free molecular flows. The TMAC is recently studied in slip and transition flow regimes [88] as required in microfluidics and nanofluidics.

The TMAC is defined as the ratio of the actual rate of tangential momentum transfer from the gas to the surface to that when gas molecules striking the surface are re-emitted as if from a gas in equilibrium at the surface temperature, and the definition is:

(29) $$\sigma_{t}\;=\;\frac{\vec{p_{ti}}-\vec{p_{tr}}}{\vec{p_{ti}}-\vec{p_{tw}}}$$

in which the subscript *t* denotes the tangential component, *p* is the average momentum of gas molecules, *i* and *r* represent the incident and reflected gas molecules, and *w* is the average parameter for those molecules who are diffusively reflected from the wall. For the wall moving at the velocity of *u_w_* , we have:

(30) $$p_{tw}\;=\;m_{g}u_{w}$$

in which *m_g_* is the mass of a gas molecule.

Since the information of the incident and reflected molecules is unknown in most cases, the TMAC is not easily predicted by theories so that the TMAC is usually obtained from experiments or numerical simulations [88]. The TMAC is quite sensitive to the gas-solid interface conditions. The impact factors on the TMAC usually include the gas-solid pairs and their interaction potentials, varieties of surface conditions such as temperature, absorbents, lattice configurations, and surface roughness. The TMAC is widely used in theoretical and practical situations and empirically chosen as unity. However, under the conditions of high temperature, high vacuum, clean surface or high speed, the TMAC is no longer unity [119,120].

##### 2.2.2.1. Experiments on TMAC

In molecular beam experiments [121,122], the incident angle and incident energy of gas molecular beams on test surfaces are fixed. The reflected distribution is gathered and both the TMAC and NMAC can be extracted with some of the results listed in Table 3. In the gas dynamics of aircrafts or space shuttles [123], the incident gas flows orient in fixed angles to the surface, and the results from the molecular beam tests are often applicable. The measured results were sometimes called beam accommodation coefficients [74]. While for gas flows in channels or tubes, the angle and energy of incident gas molecules are usually in random distributions. Thus, beam accommodation coefficients are different from those in channel or tube flows [124,125].

Since the tangential momentum exchange at gas-solid interfaces is often related to flow resistance, mass flux and slip in gas flows, experimental technique, such as the spinning rotor gauge method and flow in microchannels, were used to measure the TMAC, which have been reviewed in detail by Agrawala and Prabhu [88].

In the spinning rotor gauge method with the results listed in Table 4, a magnetized steel sphere is suspended and spinning at a high speed [129]. When the driving force is off, the sphere gradually slows down by the gas around and the TMAC can be obtained by recording the angular velocity and pressure [130,131]. Comsa *et al*. [132], Gabis *et al*. [133], Tekasakul *et al*. [134], Bentz *et al*. [135,136] and Jousten [137] have used the spinning rotor gauge method to test many monatomic and polyatomic gases as well as mixed gases on the steel surface. The method was modified by Bentz *et al*. [138] to unite the calculation model and experimental setup. As a result, the recalculated TMAC for He and Ar decreased by about 15% [134]. Lord [88,127] also used the similar method and found that the TMAC for He and Ar on the Mo surfaces were 0.20 and 0.67, while for surfaces with adsorbents, the TMAC was 0.9.

In microchannels, the TMAC is generally obtained by measuring the mass flux based on the slip models [66,139]. Arkilic *et al*. [140] found that the TMAC was about 0.8 in silica microchannels. Colin *et al*. [141], Hsieh *et al*. [142], Jang and Wereley [143,144] reported on the effects of surface roughness on the average TMAC in silica and glass microchannels. Jang and Wereley [144] further derived the relation between the averaged TMAC and the TMAC for different wall species. In addition, Huang *et al*. [145] used the pressure sensitive paint to measure the pressure distribution in microchannels. Copper *et al*. [146] studied Ar, N_2_ and O_2_ flows in carbon nanotubes and obtained same TMAC for these gases. In Blanchard and Ligrani’s experiments [147], the TMAC decreased rapidly with the increasing surface roughness on the spinning disk. Since the slip coefficients and slip models are still not consistent [66,68,88,99], the TMAC calculated according to different slip models may be different.

There are also some other methods to investigate the TMAC. In the rotating cylinder method, the Couette flows in the polar coordinate are driven by a rotating inner cylinder and a still outer cylinder, which are coaxial. When the torque is measured, the TMAC can be calculated according to the slip models [148]. Millikan [57] and his students Stacy [149] and van Dyke [58] have done many experiments to measure different TMAC for various gases on the oil surfaces in the slip flow and transition flow regimes. Kuhlthau [61] also tested the air flow and its flow resistance on Al surfaces, and the TMAC for slip flow and transition flow regimes were 0.94 and 0.74 processed by Agrawal and Prabhu [150]. Recently, Maali and Bhushan [151] performed an experimental measurement of the slip length of air flow close to glass surfaces using an atomic force microscope in dynamic mode and found that the slip length was 118 nm and the TMAC was about 0.72 with the Knudsen number varying from 0.01 to 10.

Suetin *et al.* [152] and Porodonov *et al.* [153] calculated the TMAC by measuring the relation between the relaxation time of pressure difference and the pressure in non-steady flows [88]. Shields [154–156] studied the acoustic velocity for low pressure gases and calculated the TMAC according to the relation between the acoustic velocity and velocity slip. Gronych *et al.* [157] used the viscosity vacuum gauge to measure the relative TMAC for different gas species.

The TMAC obtained from these methods mentioned above are listed in Tables 4–6 for brief comparison considering the effects of temperature and Knudsen number. The results reveal that the TMAC is quite sensitive with the surface conditions and is significantly affected by the experimental methods and surface conditions [158,159].

##### 2.2.2.2. Simulations on TMAC

As reviewed by Gak-el-Hak [13,167], the numerical means to simulate gas flows can be divided into continuum and molecular methods. The numerical methods based on the Boltzmann equations [68], the DSMC method [27,168,169] and lattice Boltzmann method (LBM) [170–173] all need given boundary conditions. The MD method [48] based on the first principle can simulate detailed gas-wall interactions if given the intermolecular potentials. Therefore, pure MD method or MD method coupled with other methods [119,174–180] are proper for studying the gas-solid momentum exchange and the accommodation coefficients.

Chirita *et al*. [181] used the Lennard-Jones (LJ) potential to simulate the incident Ar molecules on the Ni (001) surface. Various incident angles and incident energies were selected to investigate the gas molecular motions and reflected distributions near the wall. Chirita *et al*. [182] further calculated the accommodation coefficients considering the temperature effects. Finger *et al*. [183] compared the behaviors of reflected gas molecules with the experimental results by Seidl and Steinheil [126], and found that the TMAC was larger than unity due to the backscattering phenomenon as well as the effects of the adsorbent layers. Arya *et al*. [184] changed the characteristic parameters in the LJ potential to calculate the effects on the accommodation coefficients in channels. Celestini and Mortessagne [185] also simulated the Knudsen diffusion process and found that the TMAC for single molecule colliding with the wall was inversely proportional to the mean collision numbers. In these MD simulations, only the gas-wall interactions are considered and the gas-gas interactions are neglected, thus, these results are proper for highly rarefied gas flows.

Yamamoto and his collaborators [119,174–177] used the DSMC method to simulate the main flow in the center of nanochannels and the MD simulations to treat the gas-gas and gas-wall interactions near the walls. The effects of temperature, surface roughness and adsorbed molecules on the accommodation coefficients were investigated. Cao *et al*. [186] compared the velocity profiles of isothermal flows in MD simulations with the analytical solutions of the NS equations with the Maxwell slip boundary condition to extract the TMAC and found the temperature dependence of the TMAC. Spijker *et al*. [187] used the MD method to test different boundary models and calculated the accommodation coefficients in thermal conductions. Sun and Li combined the effects of temperature and adsorbed layers on the TMAC [188] and calculated the accommodation coefficients for various wall lattice configurations in smooth channels and in rough channels with nanoscale roughness. All the MD results are listed in Table 7.

According to the definition of the TMAC, one of the most important points to calculate the TMAC is to distinguish between the incident and reflected gas molecules in MD simulations. Chirita *et al*. [181] setup the escape plane in the distance of 2.36*σ_gs_* (length parameter for gas-wall interactions in the LJ potential) from the wall and recorded the information of reflected molecules on the escape plane, while Arya *et al*. [184] used 3.0*σ_gs_*. Yamamoto [119] calculated the accommodation coefficients at 8*σ_s_* (length parameter for Pt wall atoms in the LJ potential) from the wall when the gas molecules enter or leave the MD domain. Spijker *et al*. [187] set the virtual border at 2.5*σ_g_* (length parameter for gases in the LJ potential) from the wall. Sun and Li defined the incident and reflected gas molecules according to the cutoff radius in MD simulations and found that the TMAC was independent of the cutoff radius when it is larger than 3*σ_g_*.

Although the experimental and numerical researches of the TMAC have been in progress for about 50 years, the effects of many physical factors on the TMAC are still obscure. Taking the effect of Knudsen number for an example, Yamamoto *et al*. [177] studied the N_2_ gas flows in Pt nanochannels and found that the TMAC decrease with increasing Kn when there were Xe molecules adsorbed on the Pt surfaces, while the TMAC did not change on smooth surface. In gas flow experiments in microchannels, Arkilic *et al*. [140], Maurer *et al*. [94] and Hsieh *et al*. [142] also predicted the decreasing TMAC with increasing Kn. In the spinning rotor gauge method, Gabis *et al*. [133] indicated that the TMAC for He, Ne, N_2_, CH_4_ and C_2_H_6_ decreased for larger Kn, but for Ar, the TMAC decreased in the slip regime while increased in the transition regime. However, in experiments by Ewart *et al*. [166], the TMAC for Ar were almost the same for different Kn ranges. Agrawal and Prabhu [88] recommended 0.926 for monatomic gases for the entire range of Kn in their review, but it was not suitable for diatomic and other gases.

When the Knudsen number varies, the relative importance of the gas-gas and gas-wall interactions changes according to the Boltzmann equations [65,68]. Although Eckert and Drake [189] indicated that the results in the slip flow and free molecular flow regimes were relevant, the effects of Knudsen number on the tangential momentum transport were still not clarified from the above references. In addition, the selection of the value of the TMAC in slip models still depends on experience because the TMAC is quite sensitive to the surface conditions.

### 2.3. Research Insufficiency

#### 2.3.1. Non-Maxwell Reflections

From the previous two sections, the phenomena at gas-solid interfaces are so complicated because of the effects of many factors. The existing models of gas-wall interactions are incapable of describing the gas-solid transport for many non-Maxwell reflections arising from the surface roughness [190–198] and adsorbed layers above the wall.

**Figure 6.**
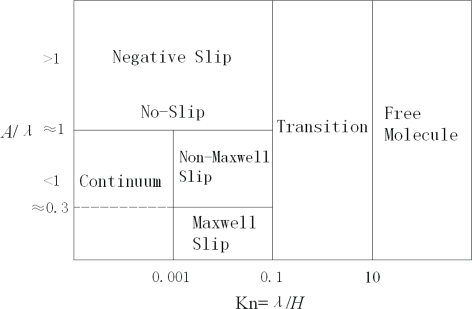
Regimes depending on both Kn and surface roughness.

The slip length is no longer linear with the mean free path when the surface roughness becomes larger above the wall, and the ratio of the roughness *A* to the molecular mean free path, *A*/*λ*, is a good criterion to use the slip boundary condition along with the NS equations. In addition, the roughness on the wall often leads to the gas molecules more accommodated with the wall so that the TMAC increases with the increasing roughness [188]. On the basis of the kinetic theory of gases, the interaction of gas molecules and a wall in the Maxwell theory is primarily based on the assumption of bounce-back behaviors, which is a linear combination of the diffusive and specular reflections. This assumption may be valid only for mathematically smooth walls. However, when the surface roughness is comparable with the molecular mean free path, this assumption is no longer rigorous. From molecular simulations [191,197,198], the backwater gases beneath the roughness interspace may play an important role in the momentum exchange between gases and solid surfaces, because the molecules impinging the backwater may undergo multicollisions inside the roughness interspace. Thus, the Knudsen layer and the wall roughness overlap. This means that the molecules can penetrate through the wall boundary region, which is quite different from an imaginary mathematically smooth surface. The molecular behaviors combining multi-collisions and permeability are responsible to the surface roughness induced non-Maxwell slippage. Regimes depending on both Knudsen number and the surface roughness can be re-categorized as in Figure 6 [197,198].

The gas molecules are easier to be adsorbed near the wall under conditions where the temperature is low [186] or the gas-wall interactions are strong [117]. When more and more molecules are adsorbed and molecular layers are formed near the walls, the molecular behaviors are consequently changed, and the momentum transport at gas-solid interfaces is dominated by both the gas-gas and gas-wall interactions. Sun and Li [117] found that the TMAC decreased with the increasing temperature for less adsorption, while the TMAC was almost independent of the temperature for quite strong gas-wall interactions.

Recently, Sokhan and Quirke [199] showed that the slip length depends strongly on the pore width for small pores tending to a constant value for pores of width larger than 20 molecular diameters for their systems, in contrast to the linear scaling predicted by Maxwell’s theory of slip. Calculating the slip length should require two material parameters: shear viscosity, which could be taken from the bulk equation of state for the viscosity, and relaxation time, which was a function of the thermodynamic state of the liquid and also depended on the pore dimensions. In these cases, the gas-solid momentum transport cannot be described by the classical Maxwell model, and more accurate boundary models are needed.

#### 2.3.2. Normal Momentum Transport

Compared with the tangential momentum transport, few researches were focused on normal momentum transport. For instance, there are many kinds of definitions for the NMAC [82,83,127, 200–206] as follows, which characterize the normal momentum transport at gas-solid interfaces:

(31) $$\sigma_{n}\;=\;\frac{\vec{p_{ni}}-\vec{p_{nr}}}{\vec{p_{ni}}-\vec{p_{nw}}}\;=\;\frac{\left|p_{ni}|\;+\;|p_{nr}\right|}{\left|p_{ni}|\;+\;|p_{nw}\right|}$$

(32) $$\sigma_{n}\;=\;\frac{\left|p_{ni}|\;-\;|p_{nr}\right|}{\left|p_{ni}|\;-\;|p_{nw}\right|}\;=\;\frac{\vec{p_{ni}}+\vec{p_{nr}}}{\vec{p_{ni}}+\vec{p_{nw}}}$$

(33) $$\sigma_{n}\;=\;\frac{\left|p_{ni}|\;-\;|p_{nr}\right|}{\left|p_{ni}\right|}\;=\;\frac{\vec{p_{ni}}\;+\;\vec{p_{nr}}}{\vec{p_{ni}}}$$

(34) $$\sigma_{n}\;=\;\frac{\vec{p_{ni}}\;-\;\vec{p_{nr}}}{\vec{p_{ni}}}\;=\;\frac{\left|p_{ni}|\;+\;|p_{nr}\right|}{\left|p_{ni}\right|}$$

in which *i* , *r* and *w* are the same as those in the TMAC definition, and *n* denotes the normal component of momentum. And when the wall temperature is *T_w_*, we have:

(35) $$p_{nw}\;=\;\sqrt{\frac{m_{g}\pi k_{B}T_{w}}{2}}$$

in which *k_B_* is the Boltzmann constant.

The definition in Equation (31) is the earliest one proposed by Liu *et al*. [127] and singular NMAC can be avoided. Defined by Equation (31), the NMAC can be larger or smaller than unity and is always near unity but it cannot be zero. While in Equation (32), the NMAC is singular at some incident angle in molecular beam experiments in which the reduced coefficients are defined [207]. In Equations (33) and (34), the singular NMAC can also be avoided but they only show the relative normal momentum of incident and reflected molecules but cannot express the accommodation with the wall.

In molecular beams, the NMAC was also calculated by Doughty and Schaetzle [125], Knechtel and Pitts [118], Seidl and Steinheil [126], and Liu *et al*. [127]. Moreover, the NMAC was investigated in MD simulations by Chirita *et al*. [182], Yamamoto [119] and Yamamoto’s colleagues [174,175,177], and Sun *et al*. [117,188]. The NMAC spans minus to larger than unity. The impact factors include temperature, absorbents and surface roughness.

## 3. Liquid-Solid Interfaces

The momentum transport behaviors at liquid-solid interfaces are quite different from those at gas-solid interfaces, though liquids and gases are both fluids from the continuum point of view. Liquids are often denser in density than gases. The average distance between molecules in gas is generally several or tens order of magnitude higher than the diameter of its molecules. However, the distance for liquids is comparable to the molecular diameter. Near gas-solid interfaces, intermolecular forces often play no role and the molecules spend most of their time in free flight between brief collisions. The momentum transport between the molecules and the solids can then be characterized by the kinetic theory well. In liquids, on the other hand, the liquid and wall molecules are in an interaction state. The concept of mean free path is not very useful for liquids. The conditions under which a liquid fails to be in quasi-equilibrium are not well defined. The intermolecular forces will play a dominant role in the momentum transport from the liquid to the wall, which cannot be characterized by existing molecular-based theory accurately thus far [208,209].

Researchers have no choice but experimental schemes and molecular dynamics simulations to detect the boundary conditions and molecular behaviors at liquid-solid interfaces. The experimental approaches require extremely accurate technologies that are capable of measuring liquid flows at nanoscale directly or indirectly. Therefore, the experimental techniques can be divided into two categories: (1) Indirect methods. The indirect methods extract the slip length at liquid-solid interfaces by measuring a specific macroscopic quantity, such as flow rate, assuming the Navier’s slip model to hold. Such methods therefore report effective slip lengths. (2) Direct methods. Recently some techniques that can trace liquid flow near a solid surface have been developed. These techniques are capable of measuring the slip velocity directly. The experimental methods can only obtain macroscopic properties. The molecular dynamics simulation method is a powerful tool to learn more detail about molecular behaviors and their physical mechanisms [47–51]. Inspired by the review outline in Ref. [31], we will focus on the research from different groups according to their investigation methods and then discuss the boundary condition dependence on different physical factors.

### 3.1. Experimental Measurements

#### 3.1.1. Indirect Methods

##### 3.1.1.1. Flow Rate through Capillaries or Microchannels (FR)

Considering a liquid flow in a capillary in a laminar state, the Navier-Stokes equations with the no-slip boundary condition gives the flow rate:

(36) $$Q_{0}\;=\;\frac{\Delta p\pi r^{4}}{8\eta l}$$

in which Δ*p* is the pressure drop down a capillary with length *l* and radius *r*, and *η* is the viscosity of the liquid. If the boundary slip is taken into account, the flow rate is:

(37) $$Q_{s}\;=\;\frac{\Delta p\pi r^{4}}{8\eta l}\;(1+\frac{4L_{s}}{r})$$

We can see that the boundary slip leads to a flow rate increase. Therefore the relative change of the flow rate arising from the boundary slip is:

(38) $$\frac{Q_{s}-Q_{0}}{Q_{0}}\;=\;\frac{4L_{s}}{r}$$

The slip length can be measured by obtaining the flow rate and pressure drop of a liquid flow in a capillary. The similar techniques can be developed for liquid flows through microchannels. Some of the experimental measurements using this method have been reviewed in Ref. [210]. We summary the experimental results [211–220] measured by this technique in Table 8. Larger flow resistances than expected with the no-slip boundary condition, perhaps negative slips, due to electrokinetic effects, flow instabilities or roughness effects, were also reported [221,222].

##### 3.1.1.2. Drainage Force (DF)

Considering a curved body moving perpendicularly toward a solid surface (steady or oscillatory), the liquid filled in the gap opposes the motion by a drainage force:

(39) $$F\;=\;-f^{*}\frac{6\pi \mu r^{2}V}{h}$$

in which *V* is the instantaneous velocity of the moving body, *h* is the minimum distance between the moving body and the surface, *μ* is the viscosity of the liquid, *r* is the radius of the moving body, and f^*^ is a correction factor when considering the boundary slip. For the no-slip boundary condition, f^*^ = 1, otherwise when there is slip, f^*^ < 1. In the symmetric case (the two surfaces have equal slip length), the correction factor is given by:

(40) $$f^{*}\;=\;\frac{h}{3L_{s}}\left[(1+\frac{h}{6L_{s}})\text{ln}(1+\frac{6L_{s}}{h})-1\right]$$

In the asymmetric case, slip is assumed to occur only on the hydrophobic surface and the correction factor is written as:

(41) $$f^{*}\;=\;\frac{1}{4}\left\{1+\frac{3h}{2L_{s}}\left[(1+\frac{h}{4L_{s}})\text{ln}(1+\frac{4L_{s}}{h})-1\right]\right\}$$

Two different experimental apparatus have been developed to measure the drainage forces: the surface force apparatus (SFA) and the atomic force microscope (AFM). The SFA technique usually uses interferometry to give the separation distance between the two surfaces. The instantaneous force is measured by attaching a spring system to the moving surface. The SFA was initially developed to non-retarded van der Waals forces through a gas, and then was extended to measure forces between solid surfaces submerged in liquids, and more recently was applied to measure slip in liquids [223–225], with results summarized in Table 9 (Group A) [225–235]. The AFM method uses a AFM cantilever to obtain the drainage force when a small sphere attached to the cantilever is driven close to a solid surface at its resonance frequency or at a fixed velocity. The experimental results measured by the AFM technique are summarized in Table 9 (Group B) [236–244].

##### 3.1.1.3. Other Techniques

**Sedimentation technique (ST):** The sedimentation speed of spherical particles is related to the boundary conditions. If the radius, *r*, of the particles is small enough, their sedimentation motion will be at small Reynolds number. The sedimentation speed with a slip length, *v*_s_, is larger than that with a no-slip boundary slip, *v*_o_, according to:

(42) $$\frac{v_{s}}{v_{0}}\;=\;\frac{1+3L_{s}/r}{1+2L_{s}/r}$$

The technique was applied in Ref. [245] with the results summarized in Table 10.

**Streaming potential (SP):** A net flow is created by imposing a pressure difference between the two ends of a capillary. The solid surfaces in contact with the electrolyte have net charges. The net liquid flow leads to a current of charges, which results in a net steady-state potential difference, termed the streaming potential, between the two ends of the capillary. The current is balanced by the conduction countercurrent in the bulk of the electrolyte. The streaming potential caused by the liquid flow with a slip length, Δ*V_s_*, is larger than that with a no-slip boundary condition as:

(43) $$\frac{\Delta V_{s}}{\Delta V_{0}}\;=\;1+L_{s}k$$

where *k* is the Debye screening parameter, which gives the typical distance close to the surface where there is a net charge density in the liquid. The technique was applied in Ref. [246] with the results summarized in Table 10.

**Droplet rolling and sliding (DRS):** In this technique small droplets move down an inclined surface under gravity. The diameter of the droplets is on the order of millimeter. The capillary number is so small that the shape of the droplets is not significantly affected by the motion. The trajectories of the water drops are recorded to analyze the droplet behaviors, rolling and sliding. Comparing with the ideal cases of solid-body roll and slid gives whether there is a nonzero effective slip or not. One of the disadvantages of this technique is that it can not measure the slip velocity quantitatively. More accurate numerical models are needed for obtaining more detailed information about the effective slip. Gogte *et al*. applied this technique to study droplet rolling and sliding which indicated slips in Ref. [247].

**Cone-and-plate torque (CPT):** In this technique a cone of radius *R* and very small cone angle *θ*_0_ rotates at angular velocity Ω over a plate. The gap between the cone and the plate is filled with a liquid. If the Navier’s hypothesis about the wall slip is considered, the degree of slip length is related to the measured torque *M* by:

(44) $$L_{s}\;=\;\frac{R\theta_{0}}{4}(1-\sqrt{\frac{8\theta_{0}}{\pi \Omega R^{3}}\;\frac{M}{\mu }\;-\;\frac{13}{3}})$$

where *μ* is the viscosity of the liquid. Choi *et al*. applied this technique in Ref. [248] and the results are also summarized in Table 10.

**Thermal motion (TM):** In Ref. [249] the TM technique to measure the boundary slip was first developed. Colloidal tracers in aqueous solution are confined between two solid silica surfaces made from a BK7 spherical lens in contact with a Pyrex plane. The thermal diffusion dynamics of the colloids is measured in this confined geometry with a fluorescence correlation spectroscopy device. The fluorescence intensity autocorrelation curve is detected to extract the residence time and the average number of beads. The residence time of the beads as a function of the confinement gives whether there is a boundary slip or not since tracer dynamics is affected by confinement, and this dependence reflects the hydrodynamic boundary conditions that apply on the solid substrates. It should be noted that the thermal motion of confined colloidal tracers allows one to characterize the nanohydrodynamics of simple liquids close to surfaces, at “zero shear rate,” and with an excellent (nanometric) accuracy.

#### 3.1.2. Direct Methods

##### 3.1.2.1. Micro Particle Image Velocimetry (μPIV)

The idea of the μPIV technique is to use small particles as passive tracers in the flow to measure the velocities of the particles with an optical method. In macroscopic fluid mechanics, the PIV method is frequently used to meansure the flow fields using particles with a radius of about micrometer or larger [250]. For microscale and nanoscale fluid flows, however, particles with nanometer scale radius are needed. Since smaller particles have larger diffusivity, results need to be averaged to observe the tracer motion. Consider a pressure driven flow between two parallel plates with a distance 2*h*. The velocity profile with a slip boundary condition is:

(45) $$u(z)\;=\;-\;\frac{h^{2}}{2\mu }\;\frac{dp}{dx}(1-\frac{z^{2}}{h^{2}}+\frac{2L_{s}}{h})$$

The PIV technique can check whether the velocities extrapolate to zero at the liquid-solid interfaces. The results obtained by this technique [251–256] are summarized in Table 11.

##### 3.1.2.2. Near-Field Laser Velocimetry Using Fluorescence Recovery (NFLV-FR)

The velocity field of small fluorescent probes can be measured close to a nearby surface. An intense laser illuminates the probes and renders them non-fluorescent. Based on monitoring the fluorescence intensity in time using evanescent optical waves, the slip length can be estimated. It should be noted that the fluorescence intensity evolves in time is due to both convection and molecular diffusion. A carful analysis is needed. As pointed out in Ref. [31], because of the fast diffusion of molecular probes, the method is effectively averaging over a diffusion length (about one micrometer), which is much larger than the evanescent wavelength.

##### 3.1.2.3. Fluorescence Cross-Correlations (FCC)

This technique was first developed by Lumma *et al*. [260] with the results summarized in Table 13. Fluorescent probes excited by two similar laser foci are monitored in two small sample volumes separated by a short distance. Cross-correlation of the fluorescence intensity fluctuations due to probes entering and leaving the observation windows allows determining both the flow direction and intensity. The measured velocities are averaged over the focal size of microscope and the characteristics of the excitation laser.

##### 3.1.2.4. Total Internal Reflection Velocimetry (TRIC)

The mechanism of the TRIC method was introduced in Ref. [261] in detail. This technique was first applied to measure boundary slips by Huang *et al*. [262,263], and then used and improved by other groups [264,265]. In this technique, an evanescent field can be created near a solid-liquid interface where total internal reflection occurs. The field intensity decays exponentially with distance away from the two-medium interface:

(46) $$I(z)\;=\;I_{0}\text{exp}(-z/p)$$

in which *I*_0_ is the intensity at the interface and *p* is the evanescent wave penetration depth. For spherical tracer particles with a uniform volumetric fluorophore distribution in an evanescent field, the particle emission intensities are an exponential function of their distances to a substrate surface. When there is a shear flow near a solid surface, the shear and near-surface hydrodynamic effects can cause a tracer particle to rotate and translate at a velocity lower than the local velocity of the fluid in the same shear plane. The apparent velocity, *Ū*, of a large ensemble of particles chosen from a normalized intensity range of *α < I^e^* */ I_0_^e^* *< β* and located in an imaging range of *h*_1_< *h* < *h*_2_ is given by the average of the local velocity integrated over the imaging range:

(47) $$\bar{U}\;=\;\frac{1}{h_{2}\;-\;h_{1}}\int_{h_{1}}^{h_{2}}U(h,\;a,\;S(h)P(h,\;\alpha \;<\;I^{e}/I_{0}^{e}\;<\;\beta )dh$$

in which *S* is the local shear rate. If there exists a slip velocity, *U*_s_, at the solid boundary, the apparent velocity of the same ensemble of particles would be:

(48) $${\bar{U}}_{app}\;=\;U_{s}\;+\;\bar{U}\;=\;L_{s}S_{wall}\;+\;\bar{U}$$

The TIRV technique uses total internal reflection of an incident laser beam to generate a highly localized illumination of the near-boundary liquid phase and relies on tracking motions of individual tracer particles to determine fluid velocity vectors in the planes parallel to a solid surface. The exponentially decaying evanescent field leads to determination of tracer particles’ positions in the direction normal to the solid surface based on their fluorescent intensities. Slip velocities and slip length can be inferred from the measured apparent velocity vectors by applying the statistical model for optical and hydrodynamic behaviors of small particles near a solid/liquid interface.

### 3.2. Molecular Dynamics Simulations

To learn more detail about molecular behaviors at liquid-solid interfaces needs first-principle based methods. Molecular dynamics simulations become one of the most powerful tools [47–51] because of the lack of molecular-based theory of liquids. Although the lattice Boltzmann method [266–270] and the atomistic-continuum (molecular-dynamics and continuum) hybrid simulations [271–277] have been extended to investigate liquid nanoflows and wetting problems in recent years, we focus on only the studies by molecular dynamics simulations here.

The MD simulations consider a set of molecules running in a region of space. The interaction between the molecules is via some potential model, such as Lennard-Jones potential. The time evolution of the molecular positions is based on integrating numerically Newton’s equations of motion. Usually the initial molecular positions are random and the initial velocities are assigned according to a Boltzmann distribution. The molecular system can be controlled to be a constant temperature by coupling with a heat bath or by rescaling the velocities of all molecules.

The problem of modeling the solid walls in liquid nanoflows is of central interest. We list four schemes in common use below. **(1). Maxwell thermal walls** [278]: Neglecting the precise microscopic structure of the walls, the reflected molecules from the boundaries experience two types of behaviors: specular and diffusive. The velocities of the reflected molecules are sampled from a Gaussian distribution:

(49) $$f(v)\;=\;\sqrt{\frac{m}{2\pi kT}}\text{exp}(-\frac{mv^{2}}{2kT})$$

**(2). Rigid lattices** [279]: Solid atoms are put at the lattice sites of the walls (most often fcc solid). During the simulation the solid atoms are constrained to remain at their lattice sites. In Ref. [279], the solid atoms were assigned a much heavier mass than the liquid molecules, *m*_solid_ = 10^10^*m*_liquid_, which allowed the atoms to move in accord with the equations of motion in very low velocities. **(3). Phantom walls** [280]: A phantom wall is kept at a constant temperature by phantom molecules modeling the infinitely wide bulk solid. The phantom wall has three layers. The first layer consists of real solid atoms whose interactions between them are via springs. The second layer is made of phantom molecules. The third layer is fixed atoms. The interactions between the phantom molecules and first-layer atoms are also via springs with a special stiffness. The connections between the phantom molecules and the fixed atoms in the third layer are via special springs and dampers. **(4). Einstein solid** [186]: This technique was first applied by Cao *et al*. [186]. The atomic structure walls are built based on the Einstein theory that the wall atoms vibrate around the face-centered-cubic lattice sites with the Einstein frequency [281]. The harmonic vibrations of the solid atoms are simulated by harmonic springs with stiffness:

(50) $$k_{stiffness}\;=\;\frac{mk_{B}^{2}T_{E}^{2}}{h^{2}}$$

in which *m* is the mass of a wall atom, *k*_B_ is the Boltzmann’s constant, *T*_E_ is the Einstein temperature of the solid, and h is the reduced Planck’s constant.

The liquid-solid interaction potential frequently used in simulations is via a modified LJ form of:

(51) $$\varphi \;=\;\varepsilon \left[{\left(\frac{\sigma }{r}\right)}^{12}\;-\;c{\left(\frac{\sigma }{r}\right)}^{6}\right]$$

where is *ɛ* an energy scale, *σ* is the atom diameter, *r* is the distance between atoms, *c* is a coefficient that allows variation of the interaction strength between liquids and solids, *i.e*., the wettability between liquids and solids. Using a simple additive model, the contact angle between the liquid and the surface can be characterized by the following formula [282]:

(52) $$\text{cos}\theta \;=\;-1+2\frac{\rho_{S}c_{LS}}{\rho_{L}c_{LL}}$$

where *ρ_S_* and *ρ_L_* are the solid and liquid density, *c*_LS_ and *c*_LL_ are respectively the liquid-solid and liquid-liquid interaction coefficients.

Two types of flow are often simulated: Coutte flow (CF) driven by the motion of the walls at constant velocities and Poiseuille flow (PF) driven by imposing a constant body force on the liquid. The boundary slips are extracted by fitting the velocity profiles using the Navier’s slip model. The MD simulation results about the boundary slip are summarized in Table 15. Early MD simulations showed that slip existed near contact lines [283,285]. More recent studies showed that slip could takes place depending on liquid-solid interactions (wettability) [282,286,288–290,295,304–310,312,313], liquid density [278,285,290,307], temperature [303], viscosity [309], pressure [282], wall roughness [289,294,296,297,301,302,305,315], surface patterns [307], shear rates [288,312,316], chain length [298], fluid motion pattern (rotation) [300], channel size [311], and also solid lattice planes [310] etc.

Two molecular mechanisms of liquid slip were found by using molecular dynamics simulations in Ref. [314]. In one form of slip, that is called a defect slip, liquid atoms hop along the solid surface from one equilibrium site to another, passing through a higher-energy transition state. The equilibrium sites compose the ground state, which is shown to exist by measuring the variance. A second mechanism, global slip, relies on the participation of the entire liquid layer. The signature of this mode of slip is parallel trajectories of all the liquid atoms, as is observed at high enough forcing.

### 3.3. Dependence on Physical Factors

According to the foregoing results which were measured experimentally or simulated numerically, we can see that many physical factors, such as surface wettability, roughness, shear rates, affect the liquid slip on a solid surface. Frankly speaking, it is quite difficult to decouple the effects of these physical factors in experiments, even in molecular dynamics simulations. Keeping this in mind we review the slip dependence on these physical factors and some theoretical models below.

#### 3.3.1. Surface Wettability

It has long been accepted that a liquid can easily slip over poorly wetted surfaces. The wettability of a surface contacting with a droplet is quantified by the contact angle [317,318]. Young’s law characterizes the relationship between the contact angle and the solid-vapor, solid-liquid, and liquid-vapor interfacial tensions as [319]:

(53) $$\text{cos}\theta \;=\;\frac{\gamma_{SV}\;-\;\gamma_{SL}}{\gamma_{LV}}$$

The surface is said to be hydrophilic if *θ* < 90° , hydrophobic if *θ* > 90° , and superhydrophobic if *θ* > 150°. A high contact angle represents a weak interaction between liquid and solid. The friction is reduced so that the liquid can slide on the solid, which causes a boundary slip. Thus far, most reports on boundary slip for liquids over solids have been related to hydrophobic surfaces as reviewed in the above sections.

In 1952 Tolstoi was the first to bridge boundary slip and surface energies (contact angles) using kinetic theory of liquids [320,321]. Later Blake extended Tolstoi’s idea to reaffirm the concept that the degree of boundary slip is related to the contact angle [322]. Based on concepts from macroscopic thermodynamics in the Tolstoi-Blake’s theory, the relation between surface energies and molecular diffusivity near a solid surface depends on the work it does for molecules to make room for themselves in the liquid, which leads to different degrees of slip at the boundaries. The mobility of liquid molecules in contact with a solid boundary can be characterized by:

(54) $$M_{s}\;=\;M_{0}\;\text{exp}[\frac{\alpha \sigma^{2}\gamma (1-\text{cos}\theta )}{k_{B}T}]$$

in which *M*_0_ is the bulk mobility of liquid molecules, *α* is a dimensionless geometrical parameter of order one representing the fraction of the microcavity area within the solid, *σ* is the molecular diameter, *γ* is the liquid surface tension, *θ* is the contact angle, *T* is the temperature, and *k*_B_ is the Boltzmann constant. It indicates that the mobility of liquid molecules at the boundary is different from the bulk liquid when the surface is not completely wetted. This gives a slip length:

(55) $$l_{s}\;=\;\sigma \left\{\text{exp}\left[\frac{\alpha \sigma^{2}\gamma (1-\text{cos}\theta )}{kT}\right]\;-\;1\right\}$$

The slip length predicted by this model is on the order of molecular diameters. The slip length vanishes for a completely wetting surface (*θ* → 0°), but increases exponentially as increasing the contact angle. The Tolstoi-Blake’s theory was recently used to quartz crystal resonators [323].

For liquid flows past ideal interfaces (atomically smooth), Bocquet and Barrat [32,324–326] derived the slip length based on the fluctuation-dissipation theorem and Green-Kubo relations from equilibrium thermodynamics. The friction, *i.e.*, the interfacial transport coefficient for momentum, is related to the integral of the autocorrelation function of the momentum flux by a Kubo-like formula:

(56) $$f\;=\;\frac{A}{kT}\int_{0}^{\infty }dt\;<\;f_{x}(t)f_{x}(0)\;>$$

where *A* is the surface area, and *f*_x_(*t*) is the tangential stress exerted by the fluid on the solid at time *t* in the x direction. A simple approximation allows one to quantify the main ingredients that characterize the friction, and gives an expression for the slip length:

(57) $$\frac{L_{s}}{\sigma }:\;\frac{D^{*}}{S_{t}c_{LS}^{2}\rho_{c}\sigma^{3}}$$

where D^*^ ; *D*_P_ / *D*_0_ , *D*_P_ is the collective molecular diffusion coefficient, *D_0_* is the bulk diffusivity, *S*_t_ is the structure factor for first molecular layer, *ρ_c_* is the fluid density at the first molecular layer, and *c_LS_* is the dimensionless liquid-solid coefficient of the LJ potential. The boundary condition is a no-slip one when the surface is completely wetting, but the slip length increases with the contact angle. When the contact angle goes to 180°, the slip length diverges as *L_s_* /*σ* ~ 1/(*π*−*θ*)^4^. This model was found to agree with MD simulations very well [282,326], and was extended to polymer solutions in Ref. [298].

More recently Huang *et al*. [313] put forward a quasiuniversal relationship between the slip length and the contact angle based on molecular dynamics simulations of water flowing on various realistic surfaces, both organic (silane monolayers) and inorganic (diamondlike and LJ models). The relationship reads:

(58) $$L_{s}\;\propto \;{\left(\text{cos}\theta +1\right)}^{-2}$$

This formula was also demonstrated to be related to the linear response theory. The slip length is related to the solid-liquid friction coefficient *f* as *L_s_* =*η*/ *f* , with *η* the shear viscosity. The friction coefficient can be given by the Kubo-like expression of Equation 56. An order of magnitude estimate for the friction can be obtained by approximating the force autocorrelation function by $\int_{0}^{\infty }dt\;<\;f_{x}(t)f_{x}(0)\;>\;\approx \;<\;f_{x}^{2}\;>\;\tau$, where *< f_x_*^2^ *>* is the mean squared surface force at equilibrium, and *τ* the relaxation time scale is given typically by: *τ* ~*σ*^2^ / *D* , with *D* the fluid diffusion coefficient. The main dependence of the friction on the fluid-solid interaction comes from *< f_x_*^2^ *>*∝ (*ε_sf_* */ σ*^2^)^2^*S*, where *ɛ_sf_* is the LJ fluid-solid energy parameter. Thus it predicts *L_s_* *= η / f* ∝ *ɛ_sf_*^−2^. Considering the Young equation and the Laplace estimate of the interfacial tensions [327], *L_s_* ∝ *ɛ_sf_*^−2^ also indicates *L_s_* ∝ (cos *θ* + 1)^−2^. All the MD results in Ref. [313] are found to roughly agree with this universal model. This model gives typical values of slip lengths ranging from a few nanometers up to tens of nanometers, in agreement with recent experiments [216,235,241,249,253], keeping in mind some exceptions [229,251].

Many experiments and molecular dynamics simulations observed apparent boundary slips for liquid flows over hydrophobic surfaces [211–220,229–243,245–249,251–260,262–265,282,286,288–290,295, 304–310,312,313], but the slip lengths obtained experimentally are much larger than the results simulated by molecular dynamics and predicted by theoretical models. Most of the slip lengths measured by the CPT technique are on the order of ten micrometers ([248] in Table 10), measured by the PIV and FCC techniques are on the order of micrometers or larger ([251,252,255,256] in Table 11, [260] in Table 13), and by the NFLV-FR technique are on the order of hundred nanometers ([257–259] in Table 12). Other experimental techniques obtained tens of nanometer of slip lengths for liquid flows past hydrophobic surfaces. The slip lengths obtained by MD simulations [282–316] are at the molecular diameter level (from one nanometer to tens nanometers, see Table 15). On the contrary, some experiments also observed slips for liquid flows over hydrophilic surfaces [229,231,233,236–240,242,243,248,258–260]. Bonaccurso *et al*. [239] demonstrated the occurrence of slip on a completely wetted silica surface which was considered to be caused by the surface roughness. Henry *et al*. [243] used different concentrations of physisorbed surfactants to make surface contact angle first increase and then decrease and found that the slip length and the contact angle were not correlated. We will discuss the effects of the nanobubbles and gas films trapped in surface roughness and patterns in detail below. From the point of these contrasting results of view, the surface wettability (contact angle) alone cannot act as a measure of slip lengths at the current stage [28,30,31].

#### 3.3.2. Surface Roughness

As pointed out in Ref. [30], no many studies concentrates on the effects of surface roughness on liquid boundary slip though surface roughness intuitively affects boundary conditions significantly. Challenges for the studies include: 1) It is difficult to control surface roughness (geometries and size) in nanoengineering situations; 2) The surface roughness may result in additional undesired changes of interface properties, such as wettability and trapped gases; 3) The uncertainty in determining the boundary position makes the interpretation of the results much complicated; 4) There is no an appropriate theoretical characterization of realistic surface roughness.

It is easy to accept that the surface roughness can distort the streamlines at the roughness scale, dissipate mechanical energy, and therefore resist the fluid flows. In 1973 Richardson [328] investigated the effects of a periodically modulated (rough) wall and showed analytically, invoking a shear stress-free boundary condition, that the no-slip boundary condition was an inevitable consequence of surface roughness. The same conclusion was also obtained by Nye [329,330] and Jansons [331]. For local no-slip condition, surface roughness shifts the position of the effective interface in the liquids, which can be regarded as effective stick-slip boundary conditions [332,333]. For liquid flows through microchannels with artificial surface roughness elements, the computational fluid dynamics (CFD) techniques could give more detailed information on velocity fields and pressure drops [333–336]. In experiments [324,325] and MD simulations [289,296,301,302], surface roughness suppressed slips were observed.

An inevitable effect of the surface roughness is the change of the surface wettability. If there are trapped gases in the gaps between the roughness elements, the interaction between the liquid and the surface will be reduced and superhydrophobic states can be obtained. The unique property of the superhydrophobicity of lotus leaves [337] and water strider legs [338] is just a typical case, which is specially called the “lotus effect” [339–348]. The roughness induced superhydrophobicity can be interpreted by the Cassie model [349]:

(59) $$\text{cos}\theta_{r}\;=\;\psi \text{cos}\theta \;+\;\psi \;-\;1$$

in which *θ_r_* and *θ* are, respectively, the contact angles of the rough and smooth surfaces, and *ψ* is the area fraction of the liquids contacting with solids. The trapped gases make *ψ* less than unity and the contact angle is increased [350,3351]. The enhancement of the surface hydrophobicity can increase the slip of liquid flows as a result. This idea was used to fabricate patterned or fractal surfaces to enlarge the slip and decrease the flow drag in microchannels [213,218,219,239,254–256,294,307,352–354]. Mathematical models and numerical analyses have also been developed in Refs. [355–360]. However, Steinberger *et al*. [361] stated that gas trapped at a solid surface could also act as an anti-lubricant and promote high friction because the liquid-gas menisci had a dramatic influence on the boundary condition. The menisci could turn the boundary conditions from slippery to sticky. They draw a conclusion that to integrate the control of menisci in fluidic microsystems designed to reduce friction was therefore essential. Govardhan *et al*. [362] found that the roughness trapped gas deceased with time, and as a result the effective slip decreased. More general drag reduction mechanisms found in nature were reviewed in Ref. [363].

It should be noted that some results show more complex slip behaviors affected by the surface roughness [244,296,297,305] because of different mechanisms of the roughness effects. Vinogradova *et al*. [244] reported that the significant decrease in the hydrodynamic resistance force of a high-speed drainage of thin aqueous films squeezed between randomly nanorough surfaces did not represent the slippage, rather than the location at the intermediate position between peaks and valleys of asperities. Cao *et al*. [305] found a dual effect of the surface roughness on the boundary slip and friction of the liquid nanoflows. In the first category, the nanostructures could enhance the surface hydrophobicity due to a nanoscale lotus effect and leaded to large velocity slips. In the second category, the nanostructures distorted the streamlines near the channel surfaces, dissipated mechanical energy, and as a result decreased the effective slips. The dual effect of the nanostructures on the rough surface resulted in a nonmonotonic dependence of the slip length on the roughness scale.

Clearly the surface roughness has significant effects on the boundary slip and flow friction of liquid flows. The above inconsistent results indicate that it is still an open question about the physical mechanisms of the surface roughness effects on the molecular momentum transport at liquid-solid interfaces.

#### 3.3.3. Shear Rate

The original Navier slip model [36] really does not mean any relationship between the slip length and the shear rate. Some experiments [230,234,235,237,244,254,257,258] and most MD simulations in Table 15 supported constant slips independent of shear rates, *i.e.*, the slip length is proportional to the shear rate. On the contrary, some experiments [229,231–233,236,239–241,243,262,263] and MD simulations [289,297,299,312,316] found shear-dependent slips, *i.e.*, the slip length is in a nonlinear relationship with the shear rate. In Ref. [216], Choi *et al*.’s experiments found explicit evidence of shear-dependent slip in hydrophobic microchannels and less clear evidences (due to the resolution limits of the technique) in hydrophilic microchannels.

Thompson *et al*. [287] provided molecular dynamics simulations to quantify the slip flow boundary condition dependence on shear rate. They found that the slip length was independent of the shear rate at low shear rates, but increased rapidly with the shear rate at high shear rates. A critical shear rate value for the slip length to diverge was obtained. Surprisingly, their results indicate that the boundary condition can be nonlinear even though the liquid remains Newtonian. Based on the MD results, Thompson and Troian [287] suggested a universal model for the slip length dependence on the shear rates:

(60) $$L_{s}\;=\;L_{s}^{0}{\left(1-\frac{\gamma^{\&}}{\gamma_{c}^{\&}}\right)}^{-1/2}$$

in which *L_s_*^0^ is the constant slip length at low shear rates, *γ*^&^ is the shear rate, and $\gamma_{c}^{\&}$ is the critical shear rate. Experiments with polymers agreed with this model [364].

Based on the experimental data in Ref. [229], the authors proposed an empirical model for the shear-dependent slip lengths [365,366]. Slip is assumed to occur locally with a constant slip length when the shear rate reaches a critical value, *i.e.*, the onset of slip at a fixed, critical shear stress. Below this critical value, the no-slip boundary condition remains valid. In this case, the slip length can be characterized by:

(61) $$L_{s}\;=\;\frac{\eta }{\tau_{c}\;-\;\tau_{c0}}u_{s}$$

in which *τ_c_*_0_ is the critical shear stress.

Lauga *et al*. [357] proposed a leaking mattress model for the shear-dependent slip lengths reported in Ref. [229]. Their idea was motivated by the observations of nanobubbles on hydrophobic surfaces [367–370]. The model considered the dynamic response of bubbles to change in hydrodynamic pressure, due to the oscillation of a solid surface. Both the compression and diffusion of gas in the bubbles decrease the force on the oscillating surface by a “leaking mattress” effect, thereby creating an apparent shear-dependent slip. The leaking mattress model is in good agreement with the experiments of Ref. [229].

High shear rates also induce viscous heating as a result of the dissipation of mechanical energy. The viscous heating then inevitably results in temperature increase and viscosity decrease of the liquids. Considering a traditional exponential law for the liquid viscosity *μ* = *μ*_0_ exp[−*β*(*T*−*T*_0_)/*T*_0_], Lauga *et al*. [210] proposed a slip length model for liquid flows in a circular capillary of radius *r*:

(62) $$L_{s}\;:\;r\frac{\beta }{T_{0}}\left(\frac{v}{k_{T}}\right)\frac{{\left(r\gamma^{\&}\right)}^{2}}{c_{p}}$$

where *T*_0_ is the reference temperature, *β* is a dimensionless coefficient of order one, *v* is the fluid kinetic viscosity, *k_T_* is the fluid thermal diffusivity, and *c_p_* is the specific heat. The shear-dependent slips observed in Ref. [248] by the CPT technique were supposed to be caused by the artificial viscous heating.

#### 3.3.4. Nanobubbles or Gas Films

Many measured apparent slips were ascribed to the presence of small amount of gas trapped or pinned on rough, patterned and/or hydrophobic surfaces [213,218,219,239,254–256,294,307,352–354]. In Ref. [245], Boehnke *et al*. reported that slip was not observed in vacuum conditions but only when the liquid sample was in contact with air in their sedimentation experiments. In Ref. [29], Granick *et al*. reported that tetradecane saturated with CO_2_ resulted in no-slip but with argon resulted in significant slip. In Ref. [252], Tretheway *et al*. calculated the slip lengths for liquid flows between two infinite parallel plates by modeling the presence of either a depleted water layer or nanobubbles as an effective air films at the walls and found the results were consistent with some experimental measurements. Using patterned surfaces to trap gases, Refs. [218,219,256] obtained apparent slips for liquid flows.

In 1983 Ruckenstein *et al*. [371] proposed the idea that liquid might flow over a gas layer (revisited in [372]). Detailed theoretical analyses showed that water between two hydrophobic surfaces was favorable to vaporize [373]. Some researchers [374–376] concerned the stability of the nanobubbles because the pressure inside the bubbles was much higher than in the surroungding solution and this should increase the gas solubility. Govardhan *et al*. [362] found that the surface roughness trapped gases deceased with time, and as a result the effective slip decreased. Considerable evidence showed that nanobubbles could exist on surfaces stably [367–370,377–382]. In Ref. [380], Borkent *et al*. performed shock wave induced cavitation experiments and atomic force microscopy measurements of flat polyamide and hydrophobized silicon surfaces immersed in water and showed that surface nanobubbles were not just stable under ambient conditions but also under enormous reduction of the liquid pressure down to −6 MPa. This implied that surface nanobubbles were unexpectedly stable under large tensile stresses.

The apparent slip length will be very large when considering liquid flows over gas films. Considering a liquid of viscosity *μ*_1_ flowing over a layer of height *h* with viscosity*μ*_2_, the apparent slip length is [383]:

(63) $$L_{s}\;=\;h(\frac{\mu_{1}}{\mu_{2}}-1)$$

For gas-water interfaces *μ*_1_/*μ*_2_ ≈ 50. As pointed out in Ref. [31], there are different situations for the effects of nanobubbles on slip length: 1) The gas in bubbles recirculates and decreases the theoretical estimate; 2) No-slip regions located between nanobubbles significantly decrease the apparent slip; 3) That bubbles are not flat decreases the theoretical prediction further. If the gas layer is in the Knudsen regime, the assumption of continuum in the gas layer breaks down, and the shear stress in the liquid is balanced by a purely thermal stress in the gas. In this case, the apparent slip length is [384]:

(64) $$L_{s}\;∼\;\frac{\mu }{\rho u_{th}}$$

in which *μ* is the viscosity of the liquid, *ρ* is the density of the gas, and *u_th_* is the thermal velocity of the gas. The apparent slip length is independent of the gas film thickness.

Exceptions should also be noted. Steinberger *et al*. [361] stated that gas trapped at a solid surface could also promote high friction because the liquid-gas menisci had a dramatic influence on the boundary condition. The menisci could turn the boundary conditions from slippery to sticky. Hampton *et al*. [385] proposed an alcohol-water exchange scheme to increase the amount of gas present on the hydrophobic surfaces in form of nanobubbles but found that a larger amount of gas increased both the long-range attractive force and the friction force due to a larger capillary bridge. More recently Hendy *et al*. [386] investigated the effective slip length for liquid flows of simple liquids over surfaces contaminated by gaseous nanobubbles. They found that, although the slip lengths over the bubbles themselves were comparable to the bubble spacing, the effects of finite slip over the bubbles might be neglected, and concluded that nanobubbles do not significantly increased slip over hydrophobic surfaces.

#### 3.3.5. Other Factors

##### 3.3.5.1. Polarity of Liquids

Cho *et al*. [242] studied the effective slippage of various nonpolar and polar liduids on alkylsilane coated glass surfaces and found that for highly polar molecules the slip length primarily depended on the dipole moment, rather than the wettability of the liquid at the surfaces, where the slip length decreased with increasing dipole moment. This result was proposed to be due to the formation of a “surface lattice structure” in the liquid between the approaching surfaces arising from dipole-dipole interactions. In addition, in Ref. [245] slip was only observed for polar liquids and in Ref. [373] the morphology of nanobubbles were found to depend on pH.

##### 3.3.5.2. Viscosity

Apparent slip lengths are thought to arise in a thin layer of liquids with lower viscosity near the wall of a smooth solid surface or in regions of higher shear next to the peaks and ridges of a rough solid [383]. However, experiments and MD simulations showed that the viscosity of simple liquids confined in very thin channels was in close agreement with [387–391] or larger than [392] the bulk value. Craig *et al*. [236] reported that the slip length increased with increasing viscosity of the liquids. Using molecular dynamics simulations, Lichter *et al*. [309] showed linear dependence of the slip length on the liquid viscosity, which was in agreement in the experimental observations in Ref. [298].

##### 3.3.5.3. Temperature

It is well understood that the liquid-solid wettability is usually temperature-dependent, and the temperature may also have an influence on the collision frequency between molecules, and thus on the momentum exchange between the fluid and the wall. In Ref. [303], Guo *et al*. reported that the slip lengths, either for normal slip or stick slip, usually decreased with increasing temperature using molecular dynamics simulations. From Lauga *et al*.’s model for the slip length (Equation 62) in Ref. [210], the slip length may be in reverse proportion to temperature. In Ref. [248], however, Choi *et al*. demonstrated that high shear rates generated viscous heating, heated the liquids, and consequently increased the slip length in their experiments.

##### 3.3.5.4. Pressure or Pressure Gradient

In Ref. [393] Tretheway *et al*. found that the slip length decreased with the increasing pressure. The no-slip boundary condition for water occurred with the absolute pressure higher than 6 atm. The results implied that an increase of pressure might decrease the sizes of the surface trapped bubbles. The pressure gradient dependence was proposed by Ruckenstein *et al*. [394] based on equilibrium thermodynamics. A pressure gradient might cause a gradient in chemical potential, hence a net force onto the liquid. The characterization of this force allowed getting the net surface velocity and estimating the slip length.

##### 3.3.5.5. Carbon Nanotubes

Direct experimental measurements [395–398] have observed high rates of water transport through carbon nanotubes using pressure-driven flows. They reported enhancements of 3–5 orders of magnitude compared with the Hagen-Poiseuille formalism. The calculated slip lengths were as large as hundreds of nanometers, even up to tens of micrometers. The following molecular dynamics simulations [399–402] also suggested that carbon nanotubes had very low surface friction with respect to fluid flow and found some physical mechanisms: (1) Water plugs formed a nonwetting contact angle inside a single-walled carbon nanotubes and formation of a vapor layer between the surface and the bulk facilitated the flow of the “bulk” water through the channel; (2) Formation of a layer of water molecules in the liquid stated on the wall of the carbon nanotubes, shielding the “bulk” molecules, which then experienced a frictionless flow; (3) Water molecules in a larger tube (about 7 nm in diameter) showed the formation of a hydrogen-bonding depletion layer near the wall, which enhanced the boundary slip greatly. The related research was reviewed in Refs. [403–405]. Likely factors, such as traveling waves [406–408], vibrations in liquids [409], and temperature gradients [410–412], should also be taken into account when investigating fluid transport inside carbon nanotubes. Though there are many open questions in this area at the moment, the rare fluid transport efficiency, as well as good electrical, optical, thermal and mechanical properties [5,413–418], makes carbon nanotubes a promising platform for microfluidics and nanofluidics engineering.

## 4. Summary and Conclusions

We have reviewed recent achievements of molecular momentum transport at fluid-solid interfaces mainly related to microfluidics and nanofluidics. The various physical factors, such as fluid and solid species, surface roughness, surface patterns, wettability, temperature, pressure, fluid viscosity and polarity, make the molecular dynamics behaviors, boundary conditions, molecular momentum accommodations, theoretical and phenomenological models quite complex. More experimental, theoretical and molecular dynamics investigations are highly required to classify the open questions in this field and to realize transport control for fluid flows at micro- and nanoscale.

Although kinetic theory seems successful to present models characterizing the molecular momentum transport at ideal gas-solid interfaces, it is still difficult to deal with more complex surface conditions and unpredictable reflections of molecules. The first issue is that there is no criterion for selecting an appropriate one from so many slip models. The second is how to quantify the introduced momentum accommodation coefficients in the slip models considering real engineering situations. The third is that theoretical characterizations need to be developed for surface molecular adsorption and trapping-desorption behaviors. Finally, the physical mechanism and law of non-Maxwell slippage still remains unclear.

Determination of the momentum accommodation coefficients is the most central mission in applying the slip models for gases flowing over solid surfaces. The researches on measuring and simulating the momentum accommodation coefficients are still uncompleted. More accurate experiments and molecular dynamics simulations are required to setup the database and develop models for different gas/solid pairs and real engineering conditions. More attentions should be paid to the normal momentum exchange at gas-solid interfaces.

The nonlinear slip models are promising to characterize rarefied gas flows inside the Knudsen layer, especially for large Knudsen number flows. The distinct nonlinear flows inside the Knudsen layer are still difficult to be modeled, whether for the constitutive relations or for the effective mean free path and viscosity, because the constitutive relations from the numerical results of the Boltzmann equations are often lack of physical meaning, and the relation between the effective mean free path and viscosity are not valid when the density is not constant along channels. Researches on nonlinear slip models for large Knudsen number gas flows are still challenging.

For molecular momentum transport at liquid-solid interfaces, the physical mechanisms for liquid slip may have two main physical images: microscopic slip at molecular scale observed by molecular dynamics simulations and phenomenal (effective) slip at macroscopic level measured by experiments. The microscopic slip length is about nanometers. The macroscopic slip length, however, spans nanometers to micrometers. Therefore, a challenge for deepening related researches is that they are incompatible in magnitude as well as in physical mechanisms. Frankly speaking, the physical mechanisms for liquid slip over solid surfaces still remain obscure at the moment.

The boundary conditions of liquid flows over solid surfaces depends on various physical factors, such as surface wettability, roughness, patterns, liquid shear rate, polarity, temperature and pressure. Molecular behaviors affected by many factors are unclear. It is necessary, but still a challenge to decouple their influences. Results obtained by different experimental techniques are often very different due to the large uncertainty in the measurements at nanoscale. Therefore, more novel experimental techniques to detect molecular behaviors near liquid-solid surfaces more accurately should be developed. More measurements and molecular dynamics simulations are needed.

The molecular dynamics simulation method is a powerful tool to detect molecular behaviors near solid surfaces, keeping in mind the long distance from the experimental measurements. The disadvantages of this method includes that it often deals with very ideal liquid-solid surfaces and the computational expense of large scale systems is heavy. It is highly desired for molecular dynamics simulations to consider real liquid and solid situations. Large scale computation using high quality computers will be useful for molecular dynamics simulations to simulate micrometer scale systems. Other particle-based techniques, such as lattice-Boltzmann and atomistic-continuum hybrid methods, are also necessary alternatives.

We are convinced that wettability and surface roughness (patterns) may be the most important factors affecting the molecular momentum transport between liquid and solid at interfaces. From this point of view, we will be able to prepare surfaces and channels in engineering with known and controllable boundary conditions, and consequently control the friction of micro- and nanoflows. Perhaps using surface patterns to trap gases and using surface coatings to artificially change the wettability are most feasible. Therefore, there leaves more open questions on formation mechanisms, physical properties and lifetime of the surface patterns trapped nanobubbles to researchers. Carbon nanotubes with rare fluid transport efficiency, as well as good electrical, optical, thermal and mechanical properties, will be a promising platform for microfluidics and nanofluidics engineering in MEMS/NEMS in the future.

## Figures and Tables

**Table 1.** Magnitudes of first- and second-order slip coefficients in linear slip models.

***C*_1_**	***C*_2_**	**References**
1	0	Maxwell [52]
1	5π/12	Schamberg (from [66])
1	−0.5	Karniadakis and Beskok [66]
1.1466	0.9756 or 0.647	Cercignani [68]
1.1466	0	Albertoni *et al*. [89]
≈1	≈0.5	Chapman and Cowling [90]
0.7252	0	Loyalka [91]
1.0299	0	Loyalka *et al*. [92]
1.11	0.61	Hadjiconstantinou [93]
1.1466	0.14	Sreekanth (from [95,96])
1	9/8	Deissler (from [66,97])
1	0.5	Hsia and Domoto (from [66,97])
1	2/9	Mitsuya [98]
1.125	0	Pan *et al*. [99]
1	0.145–0.19	Lockerby [100]
4/3	0.25	Wu (Kn < 1) [101]

**Table 2.** Expressions of first- and second-order slip coefficients in linear slip models.

***C*_1_**	***C*_2_**	**References**
$\frac{2-\sigma_{t}}{\sigma_{t}}$	0	Maxwell [52]
$\frac{2-\sigma_{t}}{\sigma_{t}}$	$-\frac{2-\sigma_{t}}{\sigma_{t}}$	Karniadakis and Beskok [66]
$\frac{2}{\sqrt{\pi }}\;\frac{2-\sigma_{t}}{\sigma_{t}}(1+0.1621\sigma_{t})$	$\frac{2}{\pi }\left(\frac{1}{2}+C_{1}^{2}\right)$	Cercignani [68]
$\frac{2-\sigma_{t}}{\sigma_{t}}\;\frac{\sqrt{\pi }}{2}(1-0.1871\sigma_{t})$	0	Loyalka [91]
$\frac{2-\sigma_{t}}{\sigma_{t}}\;\frac{\sqrt{\pi }}{2}(1+0.1621\sigma_{t})$	0	Loyalka *et al.* [92]
$\frac{2-\sigma_{t}}{\sigma_{t}}$	$\frac{9}{4\pi }\;\frac{\text{Pr}(\gamma -1)}{\gamma }$	Lockerby [100]
$\frac{2}{3}\left[\frac{3\;-\;\sigma_{t}f^{3}}{\sigma_{t}}\;-\;\frac{3}{2}\;\frac{\left(1-f^{2}\right)}{\text{Kn}}\right]$	$\frac{1}{4}\left[f^{4}+\frac{2}{{\text{Kn}}^{2}}\left(1-f^{2}\right)\right]$	Wu [101] (*f* = min[1/Kn,1])

**Table 3.** TMAC measured by molecular beam experiments.

**Authors**	**Gases**	**Walls**	**Temperature (K)**	**TMAC**
1969 [118] Knechtel and Pitts	Ar+	Au	—	0.5–0.95
		Al		0.42–0.95

1969 [125] Doughty and Schaetzle	Ar	Al with varnish	—	0.7–1.4
	N_2_			0.4–1.3

1974 [126] Seidl and Steinheil	He	Polished Cu	300	0.67–0.96
		Cu with 5μm grooves		0.96–1.16
		Cu with adsorbents		0.49–1.2
		W (100)		0.77–0.93
		Au (111)		0.68–0.87
		Glass		0.71–0.79

1979 [127] Liu *et al*.	He	Al	Room temperature	~1
		Al_2_O_3_		

1998 [128] Rettner	N_2_	C	273	> 1
		Pt	273	0.82–0.96
		Glass	293	0.80–0.98
		Disk	293	0.84–0.96

**Table 4.** TMAC measured by spinning rotor gauge experiments.

**Authors**	**Gases**	**Walls**	**Temperature(K)**	**Kn**	**TMAC**
1974 [160] Thomas and Lord	He Ne Ar Xe	polished steel rough steel	298	—	0.824, 1.040 0.918, 1.035 0.931, 1.049 0.943, 1.075

1977 [88,127] Lord	He Ne Ar Kr Xe	Mo W Ta Pt Ti	—	—	0.2 (He, Mo) 0.46 (He, Ta) 0.67 (Ar, Mo) 0.9 (contaminated)

1980 [132] Comsa *et al*.	He, Ne, Ar, Kr, Xe, CH_4_, N_2_, H_2_, O_2_, CO, CO_2_	steel	—	> 1	0.994–1.027

1996 [133] Gabis *et al*.	Ne, Ar, Kr, CH_4_, N_2_, C_2_H_6_	steel	293	0.01-1	0.83–1.01

1996 [88,134] Tekasakul *et al*.	He Ar Kr	steel	297	0.00464–0.583 0.00167–0.210 0.0013–0.163	0.8836–0.9714 0.8470–0.9381 0.8044–0.9563

1997 [88,135] Bentz *et al*.	N_2_ CH_4_	steel	294	0.00163–0.0258 0.0013–0.0215	0.83–0.89 0.98–1.11

2001 [138] Bentz *et al*.	He Ar	steel	293	Slip regime	0.8134–0.8412 0.7826–0.8005

2003 [139] Jousten	N_2_	stainless steel etched or with H_2_O covered	290–313	—	1.158–1.166

**Table 5.** TMAC in microchannels.

**Authors**	**Gases**	**Walls(Roughness)**	**Temperature(K)**	**Kn**	**TMAC**
1969 [88] Sreekanth	N_2_	brass	—	0.007–0.237	0.9317

1998 [161] Veijola *et al*.	air	Si(1 nm)	—	—	0.621–0.661
		Si(30 nm)			0.749–0.803

2001 [140] Arkilic *et al*.	Ar	Si(0.8 nm)	293	0.1–0.41	0.8 ± 0.1
	N_2_			0.1–0.34	0.83 ± 0.05
	CO_2_			0.1–0.44	0.88 ± 0.06

2001 [162,163] Sazhin *et al*.	He, Ne, Ar, Kr	Ag	—	> 100	0.71–0.92
		Ti			0.71–0.92
		Ti with O_2_ adsorbed			0.96–1.00

2003 [94] Maurer *et al*.	He	glass, Si	297-301	0.06–0.8	0.91 ± 0.03
	N_2_			0.002–0.59	0.87 ± 0.03

2003 [164] Jang *et al*.	air	glass, Si(35 nm)	298	0.00115(outlet)	0.204

2004 [141] Colin *et al*.	He, N_2_	glass, Si	294.2	0.029–0.22	0.93
				0.002–0.008	1
				0.005–0.03	0.93
				0.027–0.09	0.93

2004 [142] Hsieh *et al*.	N_2_	glass, Si(1.47 μm)	≈300	0.001–0.024 (outlet)	0.3–0.7

2004 [146] Copper *et al*.	Ar	carbon nanotubes	—	—	0.52±0.01
	N_2_				
	O_2_				

2006 [143] Jang and Wereley	air	glass (2.0 nm)	297	0.0017(outlet)	0.85
		Si(6.43 nm)			

2007 [124] Ewart *et al*.	He	Si(25.2 nm)	—	0.009–0.309	0.914 ± 0.009
	Ar			0.003–0.302	0.871 ± 0.017
	N_2_			0.003–0.291	0.908 ± 0.041

2007 [144] Jang and Wereley	N_2_	glass(2.0 nm)	295.5	0.0137 (outlet)	0.96
		SiO_2_(6.8 nm)			

2007 [145] Huang *et al*.	air	glass(0.07 μm)	—	0.018	0.90

2007 [147] Blanchard and Ligrani	He, air	Disk(10 nm)	301	0.0025–0.031	0.915, 0.885
		Disk(404 nm)			0.357, 0.346
		Disk(770 nm)			0.253, 0.145

2007 [165] Ewart *et al*.	He	Si(20 nm)	293.45–297.46	0.03–0.7	0.910 ± 0.004

2008 [166] Ewart *et al*.	He	Si(20 nm)	—	0.003–30	1.001 ± 0.019
	Ar				0.947 ± 0.010
	Xe				0.947 ± 0.015
	N_2_				0.954 ± 0.005

**Table 6.** TMAC measured by other experimental techniques.

**Authors**	**Gases**	**Walls**	**Temperature (K)**	**Kn**	**TMAC**
1949 [61] Kuhlthau	air	Forged	alloy299	0.04–0.1	0.72–1.07
		Duralumin			
				0.1–8.3	0.71–0.77 [92]
		ST-14			

1973 [88,152] Suetin *et al*.	He	glass	room temperature	slip flow regime	0.895 ± 0.004
				free molecular regime	0.935 ± 0.004
	Ne			slip flow regime	0.865 ± 0.004
				free molecular regime	0.929 ± 0.003
	Ar				
				slip flow regime	0.927 ± 0.028
				free molecular regime	0.975 ± 0.006

1974 [88,153] Porodnov *et al*.	Kr			0.00049–0.0096	0.995 ± 0.026
	Xe			0.00036–0.007	1.010 ± 0.040
	H_2_	glass	77.2	0.0011–0.022	0.957 ± 0.015
	D_2_	(0.05–1.5 μm)	293	0.0011–0.022	0.934 ± 0.006
	N_2_			0.0006–0.012	0.925 ± 0.014
	CO_2_			0.0004–0.0078	0.993 ± 0.009

1975 [154] 1980 [155] 1983 [156] Shields	He		298	—	
	Ne	Pt, Ag, W			0.375–0.96
	O_2_	rough(254 nm)			0.06–0.84
	CO_2_	adsorbents			
	N_2_				

2004 [157] Gronych *et al*.	Xe	Bronze	300.3	free molecular regime	0.90
	Ar				
					0.95
	H_2_				0.94
	He				1.0

2008 [151] Maali and Bhushan	Air	Glass	Room temperature	0.01–10	0.72

**Table 7.** TMAC calculated by MD simulations.

**Authors**	**Gases**	**Walls**	**Temperature (K)**	**Kn**	**TMAC**
1997 [182] Chirita *et al*.	Ar	Ni(001)	150	—	−0.3~0.5
			300		−0.6~0.15

2001 [119] Yamamoto	Ar	Pt(111)	300–450	0.2	0.19
	Xe				0.81

2003 [184] Arya *et al*.	LJ potential	FCC(110)	200–400	—	0–1

2005 [186] Cao *et al*.	Ar	Pt(111)	100–300	0.02-0.16	0.2–0.4

2005 [175] Takeuchi *et al*.	N2	Pt(111)	300	0.2	
		smooth			0.29–0.33
		Xe adsorbed			
					0.84–0.88

2005 [176] Hyakutake *et al*.	Ar	Pt(111)	300, 600	0.2	0.89, 0.41
	Xe				0.95, 0.80

2007 [183] Finger *et al*.	He	Cu with adsorbent layer	–––	—	0.25–1.2

2008 [117] Sun and Li	Ar	Pt	100–500	0.031–0.061	0.04–0.8

2008 [185] Celestini *et al*.	LJ potential	LJ potential	—	—	~inversed collision number

2008 [187] Spijker *et al*.	LJ potential	LJ potential	—	0.028	0.51–0.83

2009 [188] Sun and Li	Ar	Pt	100–300	0.12	0.348–0.87

**Table 8.** Summary of experimental measurements of the slip length using the FR technique.

**Authors**	**Surfaces**	**Liquids**	**Wettability**	**Roughness**	**Slip length**	**Parameter dependence**
1956 [211] Schnell	Glass + DDS	Water	--	--	1–10 μm	SRI

1984 [212] Churaev *et al*.	Quartz + TMS	Water	70–90°	--	30 nm	SRD/TD
		Mercury	115–130°	--	70 nm	SRD/TD
		CCL_4_	0°	--	No-slip	--
		Benzene	0°	--	No-slip	--

1999 [213] Watanabe *et al*.	Acrylic Resin + FAMAR	Tap water	150°	--	~240 nm	--

1999 [214] Kiseleva *et al*.	Quartz + CTA(+)	CTAB solutions	70°	--	10 nm	SRI

2002 [215] Cheng *et al*.	Glass + photoresist	Water	--	0.5 nm (pp)	No-slip	--
		Hexane	--		10 nm	SRI
		Hexadecane	--		25 nm	SRI
		Decane	--		15 nm	SRI
		Silicon oil	--		20 nm	SRI

2003 [216] Choi *et al*.	Silicon	Water	≈0°	1.1 nm (rms)	0–10 nm	SRD
	Silicon + OTS	Water	≧90°	0.3 nm (rms)	5–35 nm	SRD

2003 [217] Cheikh *et al*.	Poly(carbonate) + PVP	SDS solutions	<90°	--	20 nm	SRI

2004 [218] Qu *et al*.	Silicon (SM)	Water	>90°	--	No-slip	SRI
	Silicon (SP)	Water	130–174°	--	>20 μm	SRI/PD

2006 [219] Choi *et al*.	Silicon + SiO_2_ (SP)	Water	<90°	--	30 nm (t)	SRI/PD
					0 (t)	SRI/PD
	Silicon + SiO_2_ + Teflon (SP)		~130°	--	143 nm (p)	SRI/PD
					61 nm (p)	SRI/PD

2008 [220] Ulmanella *et al*.	Silicon	Isopropanol	--	8.5 nm	<5nm	--
		*n*-hexadecane	--		<5 nm	--
		Isopropanol	--	0.5 nm	5–30 nm	SRD
		*n*-hexadecane	--		40–120 nm	SRD

Symbols: --: unknown parameter; DDS: dimetheldichlorosilane; TMS: trimethylchlorosilane; FAMAR: fluorine-alkane-modified acrylic resin; CTAB/CTA(+): cetyltrimethyl ammonium bromide; OTS: octadecyltrichlorosilane; PVP: polyvinylpyridine; SDS: sodium dodecyl sulfate; SM: smooth; SP: surface patterned; pp: peak to peak; rms: root mean square; SRD: shear rate dependence; SRI: shear rate independence; TD: temperature dependence; PD: pattern dependence.

**Table 9.** Summary of experimental measurements of the slip length using the DF technique.

**Authors**	**Surfaces**	**Liquids**	**Wettability**	**Roughness**	**Slip length**	**Parameter dependence**
**Group A: using SFA technique**						
1985 [225] Chan *et al*.	Mica	OMCTS	--	--	No-slip	--
		Tetradecane	--	--	No-slip	--
		Hexadecane	--	--	No-slip	--

1986 [226] Israelachvili	Mica	Water	--	--	No-slip	--
		Tetradecane	--	--	No-slip	--

1989 [227] Horn *et al*.	Silica	NaCl solutions	45°	0.5 nm (av)	No-slip	--

1993 [228] Georges *et al*.	6 surfaces	9 liquids	--	0.2-50 nm (pp)	No-slip	--

2001 [229] Zhu *et al*.	Mica + HDA	Tetradecane	12°	≈0.1 nm	0–1 μm	SRD
	Mica + OTE	Tetradecane	44°		0–1.5 μm	SRD
		Water	110°		0–2.5 μm	SRD

2001 [230] Baudry *et al*.	Cobalt	Glycerol	20–60°	1 nm (pp)	No-slip	--
	Gold + thiol		90°		40 nm	SRI

2002 [231] Zhu *et al*.	Mica + OTS	Water	75–105°	6 nm (rms)	No-slip	--
		Tetradecane	12–35°	6 nm (rms)	No-slip	--
	Mica + .8 PPO	Water	85–110°	3.5 nm (rms)	0–5 nm	SRD
		Tetradecane	21–38°	3.5 nm (rms)	0–5 nm	SRD
	Mica + .2 PPO	Water	90–110°	2 nm (rms)	0–20 nm	SRD
		Tetradecane	--	2 nm (rms)	0–20 nm	SRD
	Mica + OTE	Water	110°	0.2 nm (rms)	0–40 nm	SRD
		Tetradecane	38°	0.2 nm (rms)	0–40 nm	SRD

2002 [232] Zhu *et al*.	Mica + PVP/PB	Tetradecane	--	≈0.1 nm (th)	No-slip	--
	Mica + PVA	Water	--		0–80 nm	SRD

2002 [233] Zhu *et al*.	Mica	*n*-Alkanes	Complete	--	No-slip	--
	Mica + HDA	Octane	--	--	0–2 nm	SRD
		Dodadecane	--	--	0–10 nm	SRD
		Tetradecane	12°	--	0–15 nm	SRD

2002 [234] Cottin-Bizonne *et al*.	Glass	Glycerol	<5°	1 nm (pp)	No-slip	--
	Glass + OTS	Glycerol	95°		50–200 nm	SRI
		Water	100°		50–200 nm	SRI

2005 [235] Cottin-Bizonne *et al*.	Pyrex	Water	Hydrophilic	1 nm (pp)	No-slip	--
		Dodecane	Hydrophilic		No-slip	--
	Pyrex + OTS	Water	105°	--	19 nm	SRI

**Group B: using AFM technique**						
2001 [236] Craig *et al*.	Silica + gold + thiols	Sucrose solutions	40–70°	0.6 nm (rms)	0–15 nm	SRD

2002 [237] Bonaccurso *et al*.	Mica/glass	NaCl solutions	Complete	1 nm (rms)	8–9 nm	SRI

2002 [238] Sun *et al*.	Mica/glass	1-propanol	<90°	1 nm (rms)	10–14 nm	--

2003 [239] Bonaccurso *et al*.	Silicon/glass	Sucrose solutions	Complete	0.7 nm (rms)	0–40 nm	SRD/RD
	Silicon/glass + KOH			4 nm (rms)	80 nm	SRD/RD
				12.1 nm (rms)	100–175 nm	SRD/RD

2003 [240] Neto *et al*.	Silica + gold + thiols	Sucrose solutions	40–70°	0.6 nm (rms)	0–18 nm	SRD

2003 [241] Vinogradova *et al*.	Silica/glass	NaCl solutions	Complete	0.3 nm (rms)	No-slip	SRD
	Polystyrene		90°	2.5 nm (rms)	4–10 nm	SRD

2004 [242] Cho *et al*.	Borosilicate + HTS	Octane	13°	0.3 nm (rms)	No-slip	--
		Dodecane	32°		No-slip	--
		Tridecane	35°		10 nm	--
		Tetradecane	37°		15 nm	--
		Pentadecane	39°		10 nm	--
		Hexadecane	39°		20 nm	--
		Cyclohexane	25°		10 nm	--
		Benzene	32°		50 nm	--
		Aniline	64°		50 nm	--
		Water	97°		30 nm	--
		Benzaldehyde	62°		20 nm	--
		Nitrobenzene	63°		10 nm	--
		2-Nitroanisole	70°		No-slip	--

2004 [243] Henry *et al*.	Silica/mica	Water	Complete	--	80–140 nm	SRD
	Silica/mica + CTAB	CTAB solutions	>90°	--	50–80 nm	SRD

2006 [244] Vinogradova *et al*.	Glass + Gold	NaCl solutions	90° (a)	0.5–11 nm (rms)	No-slip	SRI
			63° (r)			

Symbols: --: unknown parameter; HDA: 1-hexadecylamine; OTE: octadecyltriethoxysilane; OTS: octadecyltrichlorosilane; PPO: polystyrene (PS) and polyvinylpyridine (PVP) followed by coating of OTE; PVP/PB: polyvinylpyridine and polybutadiene; PVA: polyvinylalcohol; KOH: potassium hydroxide; HTS: hexadecyltrichlorosilane; CTAB: cetyltrimethyl ammonium bromide; OMCTS: octamethylcyclotetrasiloxane; a: advancing contact angle; r: receding contact angle; av: average; pp: peak to peak; rms: root mean square; th: polymer thickness; SRD: shear rate dependence; SRI: shear rate independence; RD: roughness dependence.

**Table 10.** Summary of experimental measurements of the slip length using other indirect techniques.

**Authors**	**Surfaces**	**Liquids**	**Wettability**	**Roughness**	**Slip length**	**Parameter dependence**
ST: 1999 [245] Boehnke *et al*.	Silica	Proanediol	≈0°	--	No-slip	--
		Proanediol + Va		--	1 μm	--
		PDMS	--	--	No-slip	--
	Silica + DETMDS	Proanediol	70–80°	--	No-slip	--
		Proanediol + Va		--	1 μm	--
		PDMS	--	--	No-slip	--

SPT: 2002 [246] Churaev *et al*.	Quartz	KCl solutions	--	2 nm (pp)	No-slip	--
	Quartz + TMS	KCl solutions	80–90°	25 nm (pp)	5–8 nm	--

DRS: 2005 [247] Gogte *et al*.	Acrylic polymer+TT Sandpaper+TT	water	156°	Smooth	--	--
			>90°	8,15 μm	Slip	--

CPT: 2006 [248] Choi *et al*.	Silicon (TOP)	Water	~10°	0.3 nm	Slip	VD
	Silicon (Teflon)		~120°	0.6 nm	Slip	
	Silicon (SP + TOP)		~0°	--	Slip	
	Silicon (SP+ Teflon)		175°	--	~20 μm	
	Silicon (TOP)					
	Silicon (Teflon)	30wt% glycerin	--	0.3 nm	Slip	VD
	Silicon (SP + TOP)		--	0.6 nm	Slip	
	Silicon (SP + Teflon)		--	--	Slip	
			--°	--	~50 μm	

TM:	Silica	Aqueous	Hydrophilic	<1 nm (pp)	No-slip	--
2006 [249]	Silica + OTS	solutions	Hydrophobic	<1 nm (pp)	~18 nm	--
Joly *et al*.	Silica + OTS		Hydrophobic	3 nm (rms)	No-slip	RD

Symbols: --: unknown parameter; DETMDS: diethyltetramethyldisilazan; TMS: trimethylchlorosilane; TOP: treated by oxygen plasma; SP: surface patterned; OTS: octadecyltrichlorosilane; PDMS: polydimethylsiloxane; pp: peak to peak; rms: root mean square; VD: viscosity dependence; RD: roughness dependence.

**Table 11.** Summary of experimental measurements of the slip length using the μPIV technique.

**Authors**	**Surfaces**	**Liquids**	**Wettability**	**Roughness**	**Slip length**	**Parameter dependence**
2002 [251] 2004 [252] Tretheway *et al*.	Glass	Water	≈0°	--	No-slip	--
	Glass + OTS		120°	0.2 nm	0.9 μm	--

2005 [253] Joseph *et al*.	Glass	Water	≈0°	0.5 nm(rms)	50 nm	--
	Glass + OTS		95°		No-slip	--
	Glass + CDOS		95°		50 nm	--

2006 [254] Truesdell *et al*.	PDMS	--	100°	--	~100 μm	SRI/PD
	PDMS (SP)		156°	--	~250 μm	SRI/PD
	PDMS (SP+ ASC)		>150°	--	~1.25 mm	SRI/PD

2006 [255] Joseph *et al*.	CNT forests	DI-water	>165°	1.7 μm	~0.47 μm	RD
				3.5 μm	~0.98 μm	
				6 μm	~1.68 μm	

2008 [256] Byun *et al*.	Glass	Water	Hydrophilic	--	No-slip	WD/PD
	PDMS		105°	--	2 μm	
	PDMS (SP)		136–145°	--	0.4–5.4 μm	

Symbols: --: unknown parameter; OTS: octadecyltrichlorosilane; CDOS: chlorodimethyloctylsilane; PDMS: polydimethylsiloxane; SP: surface patterned; ASC: aerogel solution coating; CNT: carbon nanotubes; rms: root mean square; SRI: shear rate independence; RD: roughness dependence; WD: wettability dependence; PD: pattern dependence.

**Table 12.** Summary of experimental measurements of the slip length using the NFLV-FR technique.

**Authors**	**Surfaces**	**Liquids**	**Wettability**	**Roughness**	**Slip length**	**Parameter dependence**
1999 [257] 2000 [258] Pit *et al*.	Sapphire	Hexadecane	Complete	0.4 nm (rms)	175 nm	SRI
	Sapphire + FDS		65°		No-slip	--
	Sapphire + OTS		40°		400 nm	SRI
	Sapphire + STA		25°		350 nm	SRI

2005 [259] Schmatko *et al*.	Sapphire + Al_2_O_3_	Squalane	0°	0.4 nm (rms)	30 nm	WD/MSD
	Sapphire + SiH		20°	0.4 nm (rms)	--	
	Sapphire + OTS		40°	0.3 nm (rms)	110 nm	
	Sapphire + Al_2_O_3_	Hexadecane	0°	0.4 nm (rms)	120 nm	WD/MSD
	Sapphire + SiH		20°	0.4 nm (rms)	240 nm	
	Sapphire + OTS		40°	0.3 nm (rms)	350 nm	


Symbols: --: unknown parameter; FDS: perfluorodecanetrichlorosilane; OTS: octadecyltrichlorosilane; STA: stearic acid (octadecanoic acid); rms: root mean square; SRI: shear rate independence; WD: wettability dependence; MSD: molecular shape dependence.

**Table 13.** Summary of experimental measurements of the slip length using the FCC technique.

**Authors**	**Surfaces**	**Liquids**	**Wettability**	**Roughness**	**Slip length**	**Parameter dependence**
2003 [260] Lumma *et al*.	Mica	Water	--	15 nm (pp)	0.5–0.86 μm	--
	Glass	Water	5–10°		0.6–1 μm	--
		NaCl solutions			0.2–0.6 μm	--

Symbols: --: unknown parameter; pp: peak to peak.

**Table 14.** Summary of experimental measurements of the slip length using the TIRV technique.

**Authors**	**Surfaces**	**Liquids**	**Wettability**	**Roughness**	**Slip length**	**Parameter dependence**
2006 [262] Huang *et al*.	PDMS	DI-Water	Hydrophilic	0.47 nm (rms)	26–57 nm	SRD
	PDMS + OTS		120°	0.35 nm (rms)	37–96 nm	SRD

2007 [263] Huang *et al*.	PDMS + OTS	DI-Water	120°	0.35 nm (rms)	50–110 nm	SRD
		0.1mM NaCl			30–100 nm	SRD
		1mM NaCl			50–110 nm	SRD

2008 [264] Bouzigues *et al*.	PDMS	Water	<20°	0.33 nm (rms)	−3–3 nm	--
	PDMS + OTS		95°	0.44 nm (rms)	21–29 nm	--

2008 [265] Lasne *et al*.	Glass	Water	Hydrophilic	--	No-slip	--
	Glass + OTS		90°	--	45 nm	--

Symbols: --: unknown parameter; PDMS: polydimethylsiloxane; OTS: octadecyltrichlorosilane; DI: deionized; rms: root mean square; SRD: shear rate dependence.

**Table 15.** Summary of MD simulation results on liquid boundary slip.

Authors	Solid/Liquid	Flow	Wetta-bility	Roughness	Slip length	Parameter dependence
1988 [283] Koplik *et al*.	RL/LJ	PF+CL	0–79°	No	Slip near CL	--
1989 [284] Heinbuch *et al*.	RL/LJ	PF	Complete	No	−2σ–0	--
1989 [285] Thompson *et al*.	RL/LJ	CF+CL	0–90°	No	Slip near CL	--
1989 [279] Koplik *et al*.	RL/LJ	PF	0–80°	No	0–10 σ	--
		CF				
1990 [286] Thompson *et al*.	RL/LJ	CF	<90°	No	0–2σ	WD
1992 [287] Sun *et al*.	RL/LJ	PF	--	No	Slip for frozen walls	--
1997 [288] Thompson *et al*.	RL/LJ	CF	0–140°	No	0–60σ	SRD
1999 [282] Barrat *et al*.	RL/LJ	PF+CL	90–140°	No	0–50σ	WD
		CF+CL				
2000 [289] Jabbarzadeh *et al*.	RL/hexadecane	CF	Complete	0.4-0.8 (SIN)	nm 0–10 nm	RD
2001 [290] Cieplak *et al*.	RL/LJ	PF	--	No	0–15σ	WD
		CF				
2001 [291] Sokhan *et al*.	CS/LJ	PF	--	No	1.8–10.4 nm	
2002 [292] Fan *et al*.	RL/LJ	PF	Complete	No	0–5σ	--
2002 [293] Sokhan *et al*.	CNT/LJ	PF	--	No	0–5 nm	--
2003 [294] Cottin-Bizonne *et al*.	RL/LJ	CF	110–137°	~10σ (GR)	2–57 nm	RD
2004 [295] Nagayama *et al*.	Platinum/LJ	PF	0–180°	No	0–100 nm	SRD/WD
2004 [296] Galea *et al*.	RL/LJ	CF	Complete	Atomic	−4–4σ	RD
2004 [297] Cottin-Bizonne *et al*.	RL/LJ	CF	110-137°	~10σ (GR)	0–150σ	RD
2004 [298] Priezjev *et al*.	RL/Polymer	CF	--	No	0–70σ	SRD/CLD
2004 [299] Walther *et al*.	CNT/Water	PF	~86°	No	−0.11–88 nm	CP
2004 [300] Soong *et al*.	RL/LJ or WCA	RF	--	No	0–6σ	NLS
2005 [301] 2006 [302] Yang *et al*.	TW/LJ	PF	90-140°	1.7-3.3σ (GR)	−3–8σ	RD
2005 [303] Guo *et al*.	RL/LJ	CF	--	No	−3–3σ	TD
2006 [304] Cao *et al*.	Platinum/LJ	CF	0-130°	No	−1–15σ	WD
2006 [305] Cao *et al*.	Platinum/LJ	PF	30-175°	0-2.0 nm	−5–25σ	RD/WD
2006 [306] Voronov *et al*.	CS/LJ	CF	25-147°	No	0–3.5 μm	WD
2006 [307] Cieplak *et al*.	TW/Chains	PF	0-130°	No	−4–12σ	PD
2006 [308] Li *et al*.	RL/LJ	CF	--	No	−2–8σ	WD
2007 [309] Lichter *et al*.	TW/LJ	CF	--	No	0–2.5σ	VD
2007 [310] Soong *et al*.	TW/LJ	PF	--	No	−5–30σ	LPD
		CF				
2007 [311] ^(HMDCS)^ Yen *et al*.	RL/LJ	PF	--	No	2–18σ	CSD
		CF				
2008 [312] Martini *et al*.	TW/*n*-decane	CF	--	No	0–25 nm	SRD
2008 [313] Huang *et al*.	Alkylsilane/Water	CF	40-150°	No	0–20 nm	WD
	Diamon/Water		40-150°	--		
2008 [314] Martini *et al*.	RL/*n*-decane	CF	--	No	0–2 nm	WSD
2009 [315] Sofos *et al*.	Kr/Ar	PF	--	Atomic	0–0.8σ	RD
2009 [316] Priezjev	RL/Polymer	CF	--	No	−6–24σ	SRD

Symbols: --: unknown parameters; RL: rigid lattices; TW: thermal wall; CS: carbon sheet; CNT: carbon nanotubes; LJ: Lennard-Jones fluids; WCA: Weeks-Chandler-Andersen potential; CF: Couette flow; PF: Poiseuille flow; RF: rotatiing flow; CL: contact lines; SIN: sinusoidal roughness; GR: grooves; WD: wettability dependence; RD: roughness dependence; SRD: shear rate dependence; CLD: chain length dependence; CP: configuration dependence; NLS: nonlinear slippage due to fluid rotation; TD: temperature dependence; VD: viscosity dependence; LPD: lattice plane dependence; CSD: channel size dependence; WSD: wall speed dependence (defect slip and global slip); HMDCS: Hybrid molecular dynamics-continuum simulation; No: means “no artificial roughness”.
